# Decoding Structural Disorder, Synthesis Methods, and Short- and Long-Range Lithium-Ion Transport in Lithium Argyrodites (Li_6–*x*_PS_5–*x*_Br_1+*x*_)

**DOI:** 10.1021/acs.chemmater.4c02010

**Published:** 2025-01-29

**Authors:** Hanan Al-Kutubi, Ajay Gautam, Anastasia K. Lavrinenko, Alexandros Vasileiadis, Jouke R. Heringa, Swapna Ganapathy, Marnix Wagemaker

**Affiliations:** Storage of Electrochemical Energy, Department of Radiation Science and Technology, Faculty of Applied Sciences, Delft University of Technology, Mekelweg 15, 2629 JB Delft, The Netherlands

## Abstract

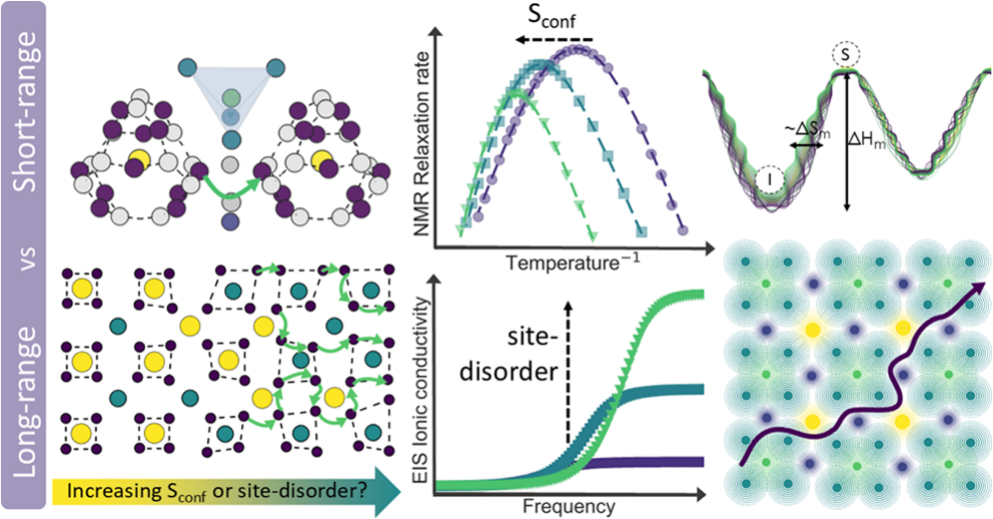

By varying the bromine content and cooling method, we are able to induce site disorder in the Li_6–*x*_PS_5–*x*_Br_1+*x*_ (*x* = 0, 0.3, 0.5) system via two routes, allowing us to disentangle the impact of site disorder and chemical composition on conductivity. Through solid-state nuclear magnetic resonance (NMR), we can explore the chemical environment as well as short-range lithium-ion dynamics and compare these to results obtained from neutron diffraction and electrochemical impedance spectroscopy (EIS). We find that the cooling method has a profound effect on the ^7^Li and ^31^P environment that cannot be explained through 4*d* site disorder alone. The configurational entropy (*S*_conf_) is used as a more complete descriptor of structural disorder and linked to distortions in both the phosphorus and lithium environment. These distortions are correlated to increased intercage movement through ^7^Li *T*_1_ spin–lattice relaxation (SLR) NMR. Further analysis of the prefactors obtained from SLR NMR and EIS allows us to obtain the migrational entropy (Δ*S*_m_). For short-range SLR movement, the Δ*S*_m_ correlates well with *S*_conf_, implying that increased intercage movement is related to distortion of the lithium cages as well as a decrease of the intercage distance. Comparison to EIS shows that an increase in short-range movement translates into increased long-range movement in a straightforward manner for slow-cooled samples. However, for quench-cooled samples, this correlation is lost. Lattice softness and phonon–ion interactions are suggested to play an important role in long-range conduction which only becomes apparent when chemical composition and disorder are disentangled. This work shows that by altering one synthesis step, the relationship between site-occupancy-based descriptors (site disorder or *S*_conf_) and lithium dynamics is changed profoundly. Furthermore, it shows that chemical composition and descriptors of site disorder cannot be seen as one and the same, as both play a role that changes with the length scale probed. Finally, it challenges the implicit assumption that increased short-range diffusivity automatically results in increased long-range diffusivity.

## Introduction

The energy transition has resulted in a focus on electrical forms of energy and their storage. Lithium-ion batteries are one of the most advanced and prevalent forms of storage of electrical energy.^1^ However, the combustible nature of the electrolyte has prompted the search for safer alternatives.^2^ Solid-state electrolytes have garnered attention as a promising alternative due to their inherent safety.^3^ Among the various classes of solid-state electrolytes, lithium argyrodites are among the most promising candidates due to their high conductivity (upward of 20 mS/cm^2^), ease of synthesis, and malleability.^4,5^

**Figure 1:**
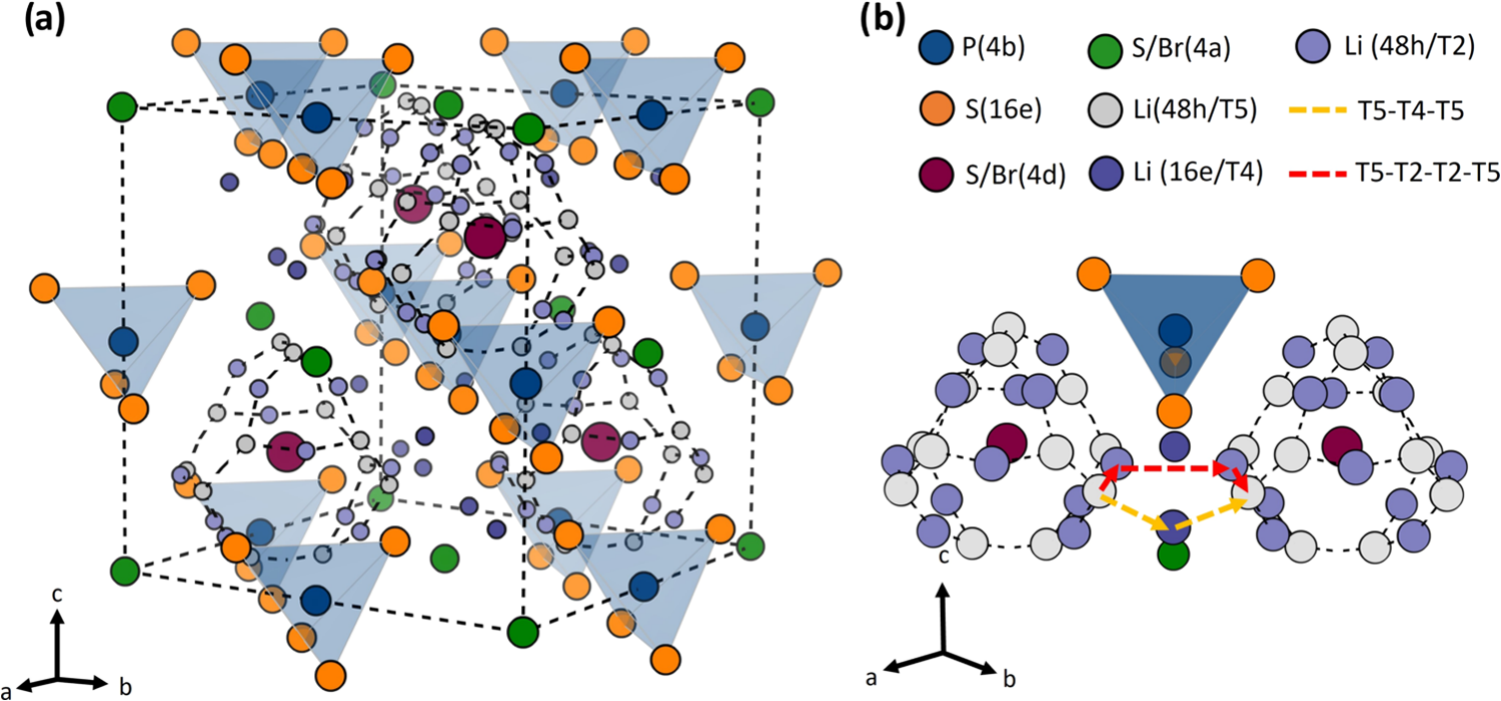
(a) Illustrated crystal structure of Li_6_PS_5_Br showing the Li cages (dotted lines) and sites. (b) Two lithium cages and the possible intercage jumps between them.

Lithium argyrodites are a crystalline class of materials with cubic symmetry and space-group *F*4̅3*m*, first mentioned by Deiseroth et al.^6^ Their general formula is Li_6_PS_5_X (X = Cl, Br, or I), and their structure is given in Figure 1. The backbone consists of PS_4_-tetrahedra, with P sitting on the 4*b* sites and S on the 16*e* sites. The remaining sulfur and halogen occupy the 4*a* and 4*d* sites. The lithium ions can occupy six different types of tetrahedral sites termed T1–T5a.^6−8^ Initially, lithium was thought to exclusively occupy 50% of the T5 sites (48*h*), forming a cage around the 4*d* site. Each cage vertex contains two T5 sites and hence one Li atom. The T5a (24*g*) site sits in between one pair of T5 sites and represents the smearing of the Li density between them.^6,9,10^ Three jumps were identified based on this model, namely, doublet jumps within one T5-pair, intracage jumps between adjacent T5-pairs within one cage, and intercage jumps.^11^ Later on, Deiseroth et al. postulated that intercage diffusion requires the T2 (48*h*’) and T4 (16*e*) sites as well.^10^ This finding is supported by band-valence and molecular dynamics simulations as well as diffraction studies on argyrodite systems.^7,12−17^ Intercage jumps can occur via two pathways: T5-T2-T2-T5 or T5-T4-T5. The latter has been found to increase intercage diffusivity as it provides a lower energy pathway.^7,12,18,19^ While all three jumps are important for fast lithium diffusion, intercage jumps are seen as the limiting step due to their high energy barrier.^11^ Facilitating intercage jumps is, therefore, considered an effective way to increase long-range ionic conductivity.

In an ordered structure, the 4*d* site is exclusively occupied by sulfur, leaving halogen on the 4*a* site. Site disorder occurs when this is no longer the case and has been shown both theoretically and experimentally to enhance the conductivity of argyrodites by facilitating intercage movement.^7,17,20−26^ Occupancy of the 4*d* site by the halogen results in a reduction of the electrostatic interaction between the 4*d* site and the surrounding Li cage. The intercage distance decreases and lithium movement is facilitated.^7,24,27^ Disorder is also correlated with increased occupancy of the T2 and T4 sites, indicating diffusion through the energetically lower T4 pathway.^24,26,28^

Increase in site disorder is conventionally achieved through halogen substitution, with the excess halogen occupying the 4*d* site, resulting in an increase in conductivity.

Gautam et al. have found that an increase in site disorder can also be achieved through altering the synthesis procedure, regardless of halogen content.^17,24^ The study has shown that materials with the same chemical composition or the same degree of 4*d* site disorder can display drastic differences in conductivity and structure.^29^ This implies that site disorder and chemical composition should neither be seen nor treated as one descriptor and that site disorder is not a complete descriptor on its own.^29^

The configurational entropy (*S*_conf_) is seen as a more complete descriptor, as it takes the site disorder of all positions into account. Increase in *S*_conf_ has been suggested to flatten the energy landscape of lithium ionic conductors, resulting in an increase in conductivity,^30^ although one recent computational study finds complex behavior.^31^ In solid-state lithium-ion conductors, increase in *S*_conf_ is almost exclusively achieved through multi-ionic doping.^32^ As with site disorder, this makes it difficult to differentiate between entropic effects and other consequences of altering the chemical composition, such as vacancy formation and change in lattice softness.

Furthermore, theoretical studies have found that, beyond structural parameters, concerted ion motion and coupling of lithium ions to the surrounding sublattice are pivotal for long-range ion movement that defines the conductivity measured with techniques such as electrochemical impedance spectroscopy (EIS) in a variety of lithium-ion conductors.^33−36^ These effects could pose a potential blind spot when trying to relate results from EIS, to structural descriptors that affect intercage movement. Conversely, EIS is also sensitive to pressure, particle morphology, and even the measurement setup, which could impede its ability to capture changes in local jump mechanics.^37−39^

In this study, we attempt to unravel the effects of chemical composition and site disorder on both long-range and short-range lithium dynamics, investigating the implicit assumption that an increase in short-range diffusion automatically results in increased long-range diffusion. We use solid-state NMR, a powerful technique to probe short-range lithium dynamics and examine the chemical environment of lithium and phosphorus within the Li_6–*x*_PS_5–*x*_Br_1+*x*_ system (*x* = 0, 0.3, 0.5).^40,41^ We compare short-range lithium intercage and intracage dynamics with long-range conductivity probed with EIS as a function of site disorder, showing that a more complete descriptor is needed. To this end, we investigated the use of configurational entropy as a descriptor. Although our materials cannot be defined as “high entropy” (*S*_conf_ < 1.5 R for all materials), a correlation between configurational entropy and conductivity has been observed for materials of lower entropy such as ours.^42^ We link the effect of increased configurational entropy to changes in the overall structure as well as distortion of the lithium substructure that are not apparent from diffraction alone. By increasing configurational entropy through chemical substitution as well as quench-cooling, we can disentangle the effects of chemical composition and structural distortion on lithium dynamics. Our results provide a link between configurational entropy and short-range lithium movement that does not hold explicitly for long-range dynamics. This trend is also observed in the entropy of migration in the conductivity prefactor, which we are able to obtain from both EIS as well as NMR. Together our results afford us a more complete picture of the effect of structural parameters on both short-range intra- and intercage movement and long-range values obtained from EIS. We show that synthesis conditions have a profound effect on short-range lithium dynamics that are not immediately apparent from EIS. We find that enhanced short-range movement does not automatically translate into enhanced long-range conductivity. Although slow-cooled samples show a clear increase in long-range ionic conductivity with increased short-range intercage movement, quench-cooled samples displayed more complex behavior. The severance of this clear link by alteration of one step in the synthesis procedure is discussed.

## Materials and Methods

The synthesis of the Li_6–*x*_PS_5–*x*_Br_1+*x*_ system was carried out through mechanochemical milling using a Fritsch Pulverisette 7 premium line instrument. The initial precursors, lithium sulfide Li_2_S (99.98%), phosphorus pentasulfide P_2_S_5_ (99%), and lithium bromide LiBr (99.99%) were purchased from Merck and Sigma-Aldrich and manually ground before ball milling. The milling process involved cycles of 10 min of milling followed by 10 min of rest over a 25 h period (10 mm ZrO_2_ media and 510 rpm). The resulting powder was then pressed into a 1.2 cm diameter pellet and placed in a quartz ampule. The ampule was pretreated at 473 K for 12 h under a dynamic vacuum to remove moisture. Ampules were sealed under vacuum and placed in a furnace for annealing. The temperature was increased at a rate of 100 °C/h, until 550 °C for *x* = 0.0 and 430 °C for *x* = 0.3 and 0.5. Two cooling methods were used: rapid quenching in liquid nitrogen and slow cooling at a rate of 5 °C/h over a 4-day period. The obtained powder was stored in an argon-filled glovebox for further characterization. The samples are referred to according to their Br content (1, 3, and 5 for *x* = 0, 0.3, and 0.5, respectively) and cooling method (SC for slow cooling and QC for quenched).

Nuclear magnetic resonance measurements were performed on a Bruker Ascend 500 magnet with a NEO console for ^6^Li and ^31^P and a Bruker UltraShield 300 magnet with an Avance console for ^7^Li. For the ^6^Li, ^31^P, and ^7^Li measurements, a 4 mm rotor with a zirconia cap was employed together with a 4 mm triple resonance probe. Rotors were dried under dynamic vacuum overnight, filled, and closed in an argon-filled glovebox to avoid degradation. The ^6^Li and ^7^Li spectra were referenced vs LiCl in water and the ^31^P spectra vs 85% H_3_PO_4_ in D_2_O (Sigma). The Larmor frequencies were 73.6, 202.457, and 116.642 MHz for ^6^Li, ^31^P, and ^7^Li, respectively. For the spectra given in the text, a one pulse/Bloch decay experiment was used for the ^6^Li and ^31^P MAS spectra and a solid-echo experiment was used for the ^7^Li static spectra. Longitudinal relaxation in the laboratory frame (*T*_1_) measurements were performed by using a saturation-recovery sequence. For each sample, the 180° pulse length and spin–lattice relaxation time (*T*_1_) were determined before acquiring the final spectra. The pulse length was the 180° pulse length divided by 2, and the delay between scans was 5*T_1_ to ensure full relaxation. For the ^6^Li and ^31^P spectra, a MAS rate of 5 kHz was employed to minimize dipolar interactions. For ^6^Li, pulse lengths varied between 4.6 and 4.8 μs and delays were 40 or 50 s for 8 scans. For ^31^P, pulse lengths were between 3.5 and 5.3 μs, delays were 50 s with at least 32 scans obtained. For ^7^Li, pulse lengths varied between 2.95 and 3.75 μs. Analysis of the spin–lattice relaxation (SLR) data was done using Bruker TopSpin, sSnake,^43^ and a custom python script. Deconvolution of the ^31^P and ^7^Li peaks was performed using MestreNova.

Temperature-dependent electrochemical impedance spectroscopy measurements (EIS) were conducted using an Autolab PGSTAT and EC10 M impedance analyzer connected to a Fryka climate chamber. A frequency range of 10 MHz to 1 Hz and a perturbation amplitude of 0.01 V were used. The capacitance of the wires used was determined beforehand by replacing the sample cell with a 10 MΩ resistor.

Density functional theory (DFT) calculations based on the Perdew–Burke–Ernzerhof functional for solid-state systems (PBEsol)^44,45^ within the Vienna Ab initio Software Package (VASP)^46^ were performed. Structure optimizations were conducted with an energy cutoff of 340 eV in 2 × 1 × 1 argyrodite supercells. The experimentally determined S- and Br- site occupancies for the six materials were used to create six corresponding structures.^29^ A Li_6_PS_5_Br-structure with no site disorder was also analyzed. For each system, the lowest-energy configuration was identified from various random S/Br arrangements and subsequently studied with *ab initio* molecular dynamics (AIMD) in the NVT ensemble at 650 K to obtain decent statistics. The selected time step was 2 fs for a total computational time of 150 ps. The analysis was conducted according to the method of de Klerk et al.^47^ Three positions were defined, namely, 48*h* (T5), 16*e* (T4), and 48*h*’ (T2) in accordance with the lithium occupancy observed with neutron diffraction.^29^ The simulation was divided into 5 parts to obtain the standard error.^11^

## Results and Discussion

**Figure 2:**
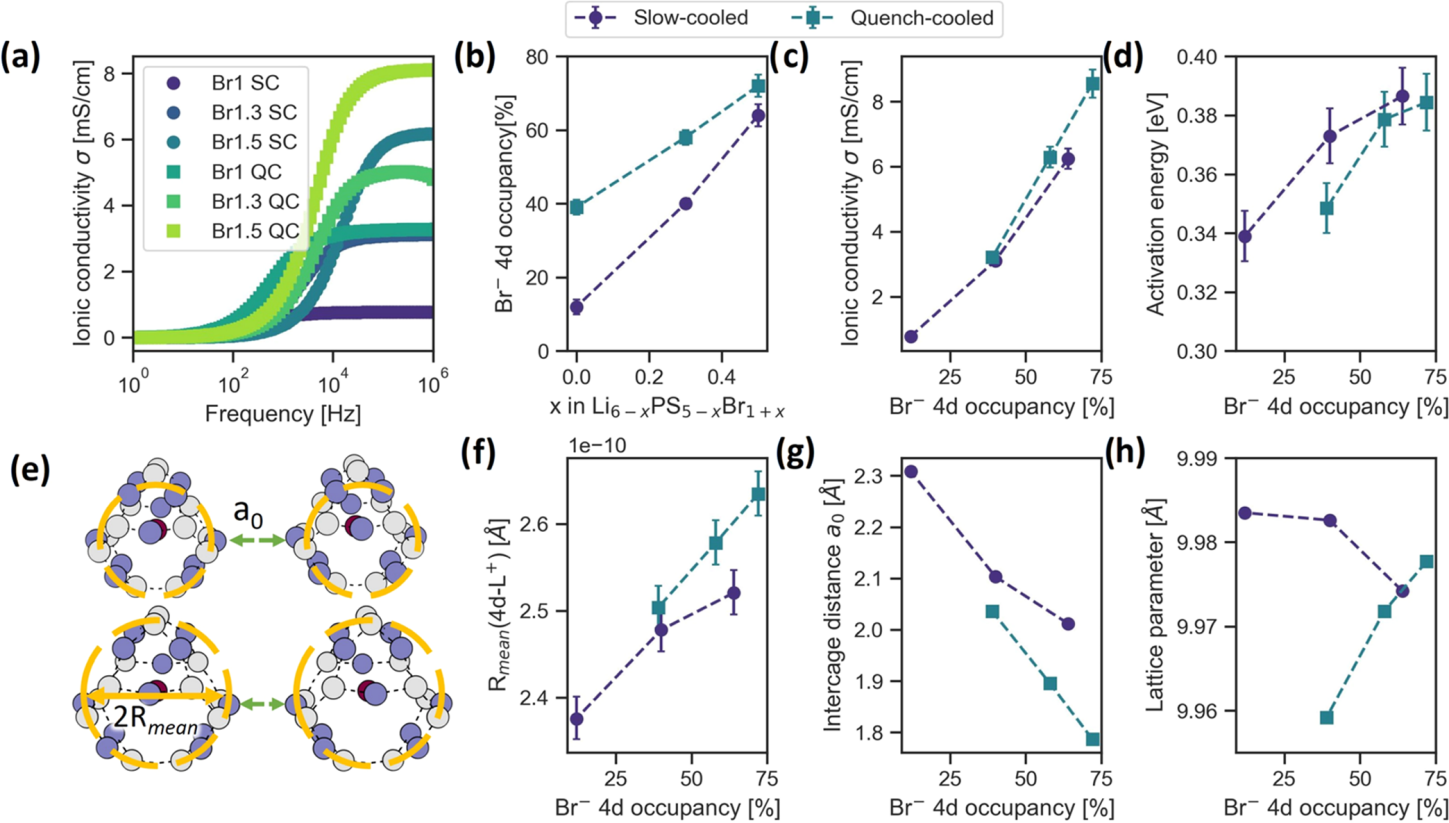
Results obtained by Gautam et al.^29^ showing (a) the conductivity as a function of applied frequency from EIS measurements for each sample, (b) the 4*d* site disorder as a function of Br content, (c) the ionic conductivity from EIS as a function of site disorder, (d) the activation energy obtained from EIS as a function of Br content, (e) the effect of cage expansion on *R*_mean_ and the T2–T2 distance, (f) the *R*_mean_ values, and (g) the T2–T2 distance obtained from neutron diffraction. (h) Lattice parameters obtained from X-ray diffraction. Data is taken from ref (29).

Previous work by Gautam *et al.* focused on diffraction and EIS to elucidate the relationship between site disorder and ionic conductivity in the Li_6–*x*_PS_5–*x*_Br_1+*x*_ system.^29^ The site disorder is defined as the percentage occupancy of Br^–^ on the 4*d* site and was varied by increasing the Br content (*x* = 0, 0.3, 0.5) as well as the cooling method used after synthesis. Samples were either slowly cooled over a span of several days or quenched immediately by submersion in liquid nitrogen. The results of the work of Gautam et al. are summarized in Figure 2, focusing on the effect of site disorder. For the X-ray and neutron diffraction patterns and a comparison between the effects of site disorder and *x* value, see Supporting Information (SI) section 1. No correlation between the peak width and the degree of disorder was observed (see SI section 1). The cooling method and chemical composition have a clear effect on the EIS response given in Figure 2a. Figure 2b shows that increasing the Br content results in increased site disorder as additional Br displaces sulfur at the 4*d* site. Quenching increases site disorder further by “freezing in” the higher site disorder present at the synthesis temperature, resulting in higher room-temperature disorder at the same Br content.^17^ The ionic conductivities and corresponding activation energies are expected to depend on both the Br content and the site disorder of the samples. The ionic conductivity was found to correlate more strongly with site disorder, as shown in Figure 2c. Samples with similar site disorders are found to have similar conductivities, regardless of Br content. This is less the case for the activation energies (Figure 2d), showing a difference, especially at low bromine 4*d* occupancy. An increase of the activation energy with increasing conductivity is observed, congruent with the Meyer–Neldel rule.^48^
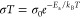
1The ionic conductivity can be defined using eq 1, with *E*_a_ being the activation energy and σ_0_ being a prefactor that contains an entropic term (vide infra for a more detailed discussion). The Meyer–Neldel rule predicts an increase in the prefactor with increasing *E*_a_ resulting in an increase in conductivity. Some studies have seen the opposite behavior for other doped argyrodites, such as Li_6–*x*_PS_5–*x*_Cl_1+*x*_,^20,25^ Li_6–*x*_PS_4.5_Cl_*x*_Br_1.5–*x*_^42^, and Li_6–*x*_PS_5–*x*_Br_1+*x*_^27^ synthesized via a different procedure and different conditions. A systematic study of lithium-ion conductors by Gao et al.^48^ predicts classical Neldel–Meyer behavior for materials with a Meyer–Neldel energy (MN energy) of less than 26 meV, which is the case for our materials (see SI section 2) and the opposite behavior for materials that show an MN energy above 26 meV, seen in the studies mentioned. Yelon et al. define the Meyer–Neldel energy as the average energy of the excitations responsible for conduction, multiplied by a coupling constant.^49^ Hence, it is possible that differences in chemical composition and synthesis method can affect the average excitation energy as well as the coupling constant, resulting in a different MN energy and hence different behavior.

Structural analysis using neutron- and X-ray diffraction revealed that an increase in site disorder results in an expansion of the lithium cage surrounding the 4*d* site as well as increased occupancy of the T2 and T4 sites. This is shown in Figure 2f, where *R*_mean_ denotes the average radius of the Li cage and is found to correlate well with site disorder. The effect of the cooling method is also apparent for the lattice parameters given in Figure 2h, which show opposite trends for slow-cooled and quenched samples. These observations have been explored previously and are due to the occupancy of the 4*a* site and distribution of lithium and its vacancies.^29^ The intercage distance, taken as the distance between two 4*d* sites minus twice the *R*_mean_ shows a clear decrease in both cases, yet also a clear difference between cooling methods. The results show that long-range ionic conductivity correlates well with site disorder, whereas its activation energy does not. Furthermore, the intercage distances display clear differences depending on cooling method. The long-range ionic conductivity seems to be inversely correlated to the intercage distance although a slight deviation is observed. Between the structure and long-range conductivity sits the realm of short-range conductivity, defined by the intercage and intracage jumps. To probe the interplay between structure and lithium dynamics further, solid-state NMR was used to probe short-range lithium movement as well as the local structure in more detail.

### Ion Dynamics and Energetics

Spin–lattice relaxation nuclear magnetic resonance (SLR NMR) was used to probe the energetics of short-range lithium diffusion. NMR probes the nuclear spin states of materials through the application of radio frequency pulses. Under thermodynamic equilibrium, spins are distributed over the available energetic spin states of a material according to a Boltzmann distribution. The radio frequency pulse shifts the system away from thermodynamic equilibrium. Relaxation to equilibrium occurs via either spin–spin or spin–lattice relaxation, each with its own characteristic relaxation time (*T*_2_ and *T*_1_, respectively). Spin–lattice relaxation occurs via fluctuations in the local magnetic field at the characteristic Larmor frequency (ω_0_) of the nuclei measured. These fluctuations depend on the surroundings and change as the nuclei diffuse. The speed of this change can be quantified by the decay of the autocorrelation function of these fluctuations. The decay will depend on the rate of diffusion and is characterized by the correlation time (τ_c_). This time differs by an approximate order of magnitude from the residence time (τ) and is assumed to follow an Arrhenius-type behavior (eq 2) where *E*_a_ is the activation energy and τ_0_ is a prefactor.^50^ In crystalline solids, the jump rate is defined as the inverse of the residence time.
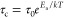
2

The modified Bloembergen–Purcell–Pound model (BPP model), given in eq 3 defines the relationship between the average correlation time and the measurable *T*_1_.^51^ The classic BPP model for three-dimensional (3D), uncorrelated motion predicts that the *T*_1_-rates will display a symmetric curve as a function of temperature, with a maximum at 1/*τ*_c_ ≈ ω_0_. The constant *C* determines the height as well as the width of the curve and is proportional to the extent of dipolar and quadrupolar coupling.^51,52^ The two flanks on either side of the maximum are the high- and low-temperature flanks. For systems that show correlation effects or Coulombic interactions, the curve will display an asymmetry, with a smaller slope for the low-temperature flank compared to the high-temperature flank.^53,54^ The β value quantifies this asymmetry by taking into account the possibility of multiple correlation times^53,54^ and is defined in eq 4. Here, *E*_a_ is the activation energy obtained from the BPP fit and *E*_a_^LT^ corresponds to the activation energy associated with the low-temperature flank. For a symmetric curve, β is 2.
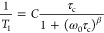
3
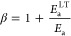
4

In argyrodite systems, asymmetry is often encountered and is thought to be a manifestation of the different energy barriers for intercage and intracage jump processes.^21,53,55−57^ The low-temperature flank is associated with localized movement, namely, doublet and intracage jumps, whereas the high-temperature flank pertains to intercage jumps as well. Relaxometry *T*_1_ measurements are sensitive to jumps that occur in the time scale of the Larmor frequency (ω_0_ = 116 MHz in this case) and probe the jumps that occur within 1/ω_0_. When very few intercage jumps occur within this time frame, both flanks represent local movement and a symmetric curve is obtained. The appearance of intercage movement at higher temperatures results in an asymmetric curve.

**Figure 3:**
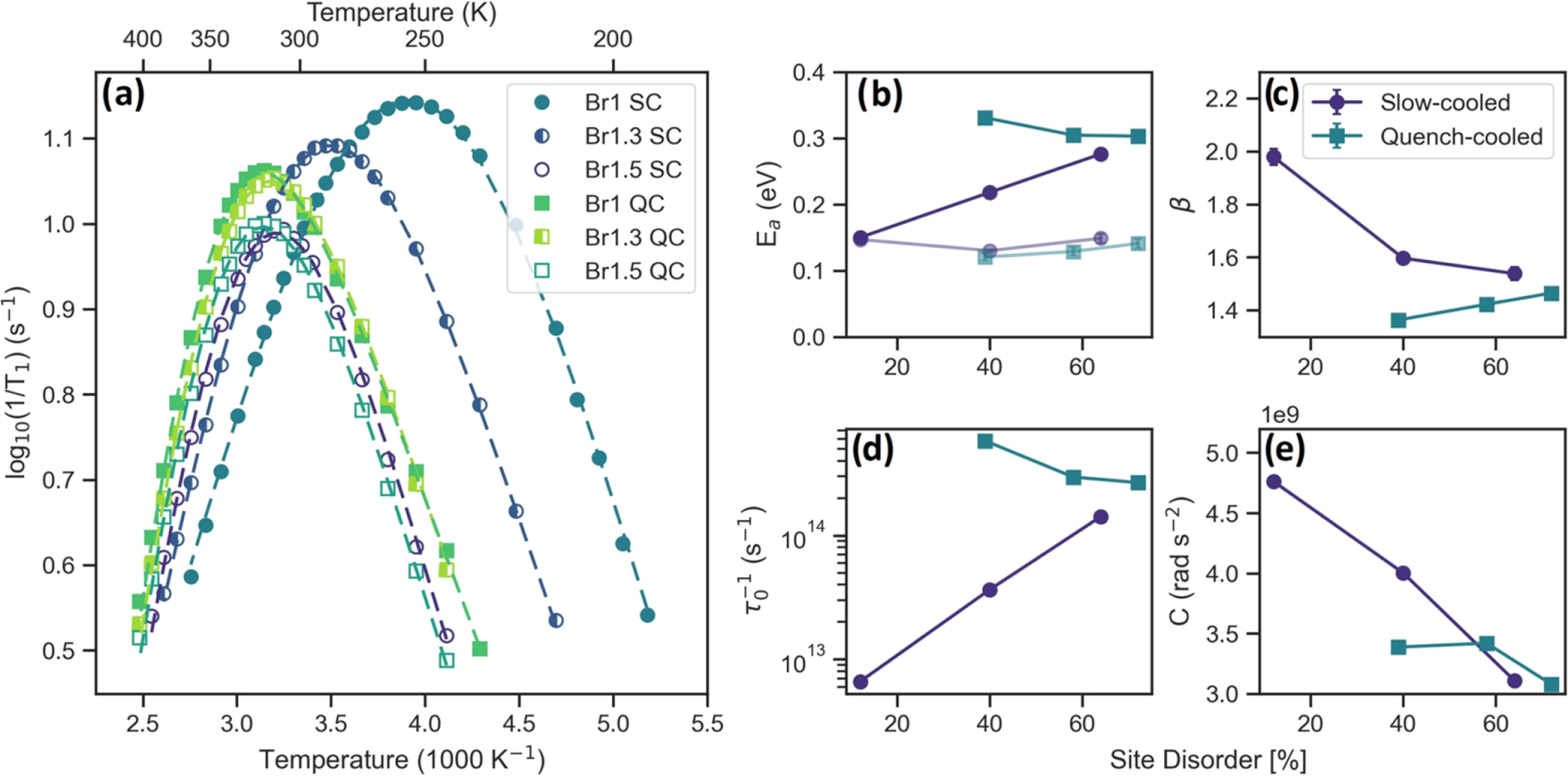
(a) Spin–lattice relaxation rates (1/*T*_1_) measured using a saturation-recovery sequence and the fit (dashed lines) using the modified BPP model for the slow-cooled and quench-cooled samples as a function of temperature, (b) the activation energy (*E*_a_) from the modified BPP fit (solid/solid line) and the low-temperature BPP *E*_a_^LT^ (transparent/dashed line) obtained using eq 4, (c) the asymmetry parameter, (d) prefactor, and (e) *C* values obtained from the modified BPP fit as a function of site disorder obtained from diffraction measurements.^29^

The *T*_1_ relaxation times as a function of temperature as well as the values obtained from the modified BPP model fit are given in Figure 3.

For slow-cooled samples (SC), the *T*_1_ curves, given in Figure 3a display a single peak that shifts to higher temperatures with increasing bromine content and site disorder, meaning that the temperature at which the jump rate approximates the Larmor frequency (ω_0_ ≈ 116.6 MHz) increases. The Larmor frequency is mainly defined by the nucleus and differs only slightly for these materials, implying a lower jump rate at a given temperature, in contrast to the increased conductivity observed with EIS.

The activation energies obtained from fitting the relaxation curves are given in Figure 3b as a function of the site disorder and are similar to the literature.^21,58−61^ For the slow-cooled samples, an increase in the activation energy is observed. This is explained by a decrease in asymmetry parameter β (Figure 3c), which can be seen as a measure of the degree of intercage jumps. For Br1 SC, an almost symmetric curve hints toward a lack of intercage jumps. Increasing the Br content results in a decrease in β and hence more intercage movement. These jumps possess a larger activation barrier, resulting in an increase in *E*_a_, a decrease in the overall jump rate, and a shift of the curve maxima to higher temperatures.

For quench-cooled samples, the opposite trend is observed. The Br1 QC sample shows the highest activation energy and asymmetry. This implies that intercage movement occurs readily in Br1 QC. An increase in the Br content and site disorder slightly lowers the activation energy and increases the asymmetry parameter. The asymmetry depends on the activation energy of the low-temperature flank (*E*_a_^LT^) and the high-temperature flank. A slight increase in *E*_a_^LT^ is observed for both cooling methods, possibly correlated with the cage expansion observed with neutron diffraction, which reduces the rate of intracage and doublet jumps. This, combined with a slight decrease in the overall *E*_a_, could result in a slightly higher β value.

Information about the Li environment involved in relaxation can be inferred from the *C* value (Figure 3e). Spin–lattice relaxation of lithium can occur either through dipolar- or quadrupolar interactions with the surrounding lattice. The extent of these interactions and the relative dominance of one over the other depends on the chemical environment.^62^ Dipolar interactions depend on the gyromagnetic ratio of the surrounding nuclei and are inversely proportional to the distance between the nuclei. Quadrupolar interactions depend on the symmetry of the lithium environment, with a larger asymmetry resulting in a larger degree of coupling. Spin–lattice ^7^Li relaxation of lithium argyrodites is known to be due to a mixture of these interactions. As can be seen in Figure 3e, an increase in disorder and Br content results in a decrease in the *C* value, implying a change in the lithium environment. Line shape analysis (vide infra) shows that quadrupolar coupling increases with site disorder. The decrease observed in Figure 3e could, therefore, be due to decreased dipolar interactions. In lithium argyrodites, this decrease has been correlated to an increased occupancy of the non-T5 sites.^26,56^ As these sites are further away from the 4*d* site and other lithium species, heteronuclear dipolar coupling between lithium and the 4*d* species and homonuclear coupling between lithium would decrease, resulting in a smaller *C* value. Calculation of the Li–Li distance using the *C* value gives results of a similar order of magnitude as expected (see SI section 3) but a different trend to Figure 2g. The Li–Li distances increase with increasing site disorder and are therefore not a reflection of the intercage distance but of the average Li–Li distance experienced by the lithium nuclei involved in relaxation. For slow-cooled samples, this trend indicates that the non-T5 sites associated with intercage movement play a more prominent role in relaxation with increased site disorder, corroborating the increase in intercage movement implied by the decrease in β and increase in *E*_a_. For quench-cooled this explanation implies that intercage movement occurs readily and increases slightly with increasing site disorder.

The SLR results indicate that both the cooling method and the Br content have an impact on the number of intercage jumps. The effect of the increased bromine content also differs per cooling method. For the slow-cooled samples, increased bromine content results in an increase in the number of intercage jumps and an increase in the activation energy. For quench-cooled samples, intercage movement occurs readily regardless of bromine content, though it decreases the activation barrier. A similar trend is seen in the MD simulations of these materials. The AIMD simulations were conducted at 650 K to obtain decent jump statistics as is common for lithium argyrodites, particularly when looking at the effects of site disorder.^11,12,22^ Previous studies indicate good agreement between simulations and SLR data.^16,63,64^

**Figure 4:**
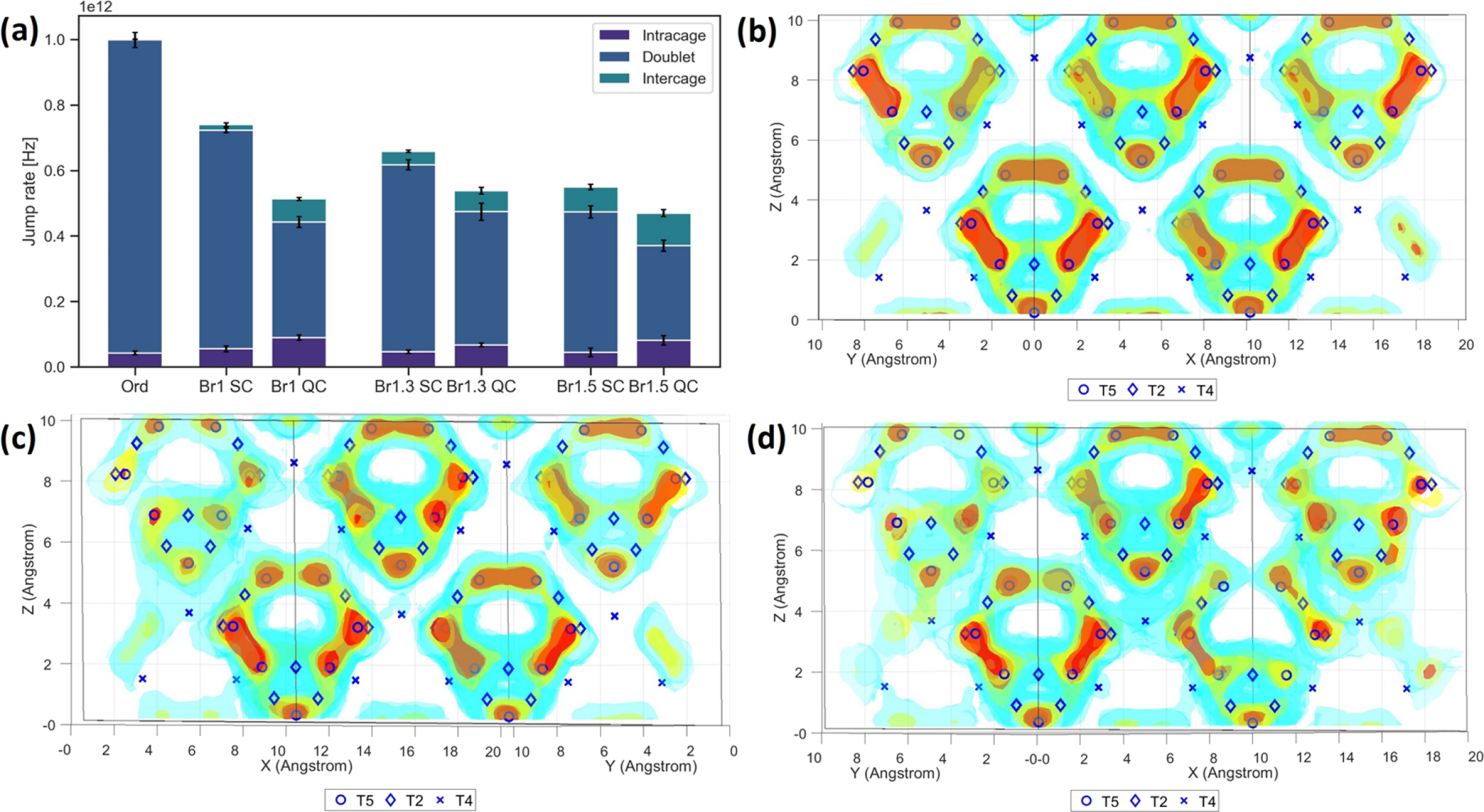
Results obtained from MD simulations at 650 K showing (a) the jump rates of the three jump types for a system with no site disorder (“Ord”) and systems with equal site disorder to the experiment; (b) the lithium densities for Li_6_PS_5_Br with no site disorder; and structures representing (c) Br1 SC and (d) Br1 QC.

Figure 4 shows results from MD simulations of a system with no site disorder (“Ord”) and systems with 4*d* and 4*a* occupancies for bromine and sulfur equal to those obtained experimentally for the investigated samples. The lithium density plots given in Figure 4b,c,d show the effect of disorder on Li_6_PS_5_Br (for the lithium density map of all structures, see SI section 5). For a structure with no site disorder (Figure 4b), lithium sits in cages surrounding the 4*d* site and no intercage jumps occur during the simulation time. A similar situation is seen for the Br1 SC system (Figure 4c) albeit with a slight increase in intercage movement. The more homogeneous distribution of lithium density for the Br1 QC system (Figure 4d) indicates an increase in lithium diffusion between cages, as well as an increase in the occupancy of the non-T5 sites. This is also seen in the jump rates given in Figure 4a. For the slow-cooled samples, an increase in site disorder and Br content correlates with an increase in intercage jumps. Intercage jumps require more time as they span a longer distance and have a higher energy barrier, resulting in a decrease in the total jump rate within the simulation time. For the quench-cooled samples, the Br1.5 QC system displays the largest amount of intercage jumps and the lowest total jump rate. The increase in intercage jumps implies that the decrease in *E*_a_ and asymmetry seen for the quench-cooled samples in Figure 3 are not due to a decrease in the amount of intercage movement but due to a decrease in the overall energy barrier. Interestingly, the Br1 QC has a much higher intercage jump rate than Br1.3 SC, despite similar degrees of site disorder, mirroring the results from SLR NMR. Its intercage jump rate is also similar to Br1.3 QC, despite possessing a lower degree of site disorder.

The AIMD simulations agree well with the SLR results, indicating that for the slow-cooled samples, an increase in bromine content and site disorder result in an increase in intercage movement. For the quench-cooled samples, this trend is less prominent, as the Br1 QC sample displays a significant degree of intercage movement. Although these results follow a somewhat similar trend to the intercage distance in Figure 2g, neither this distance nor the bromine content or site disorder can fully explain the trends observed and why they differ from results obtained from EIS. A deeper look into the structure done in the next section might shed more light.

### Structure–Property Relationship

**Figure 5:**
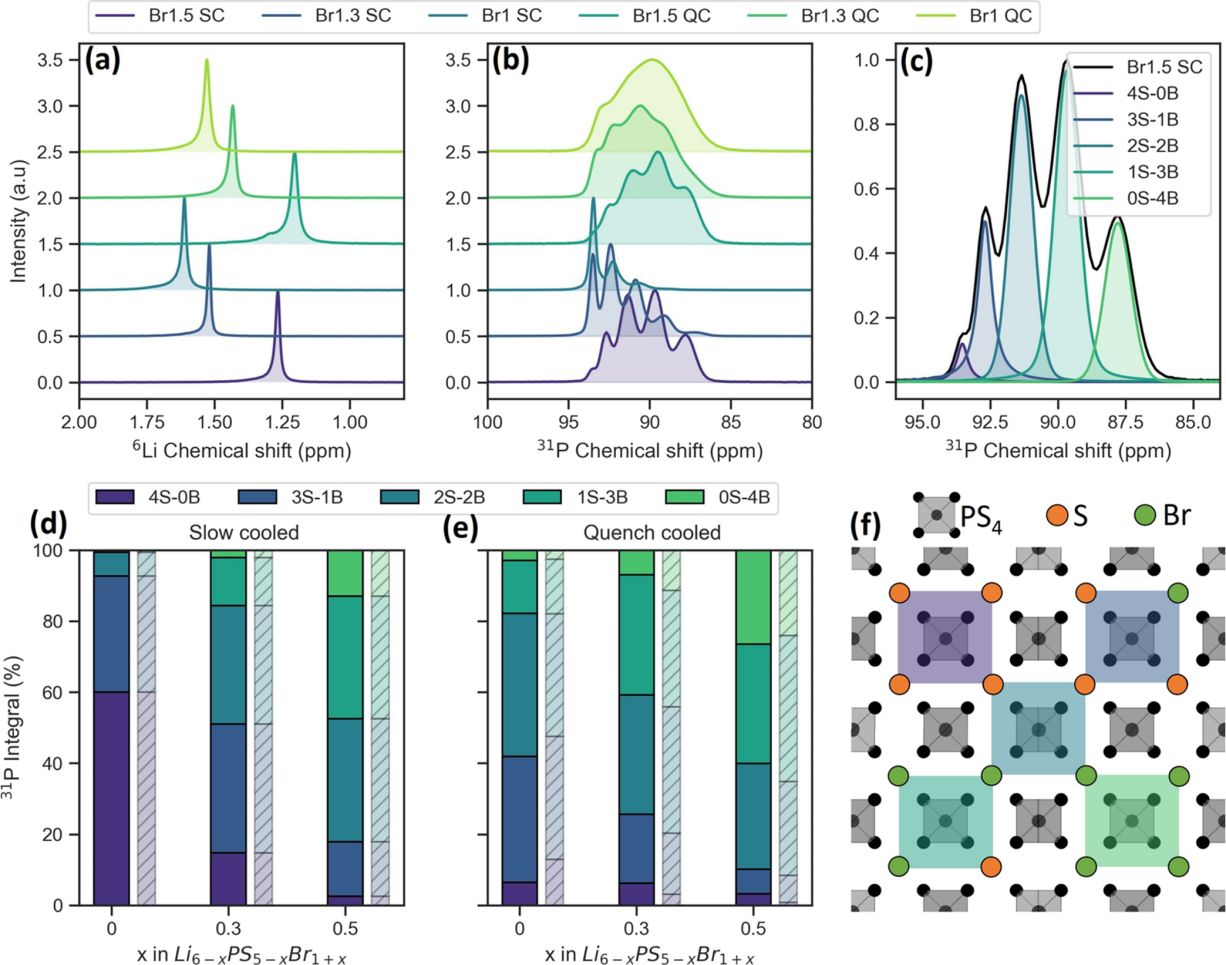
(a) ^6^Li and (b) ^31^P NMR spectra at MAS rate of 5 kHz for all samples measured at 293 K; (c) a deconvolution of the Br1.5 SC ^31^P spectrum showing five environments, the integral fractions (solid) and the theoretical prediction (hashed) of the five environments for the slow-cooled (d) and quench-cooled (e) samples; and (f) illustration of the PS_4_-tetrahedra surrounded by S/Br in the 2nd coordination shell leading to the five ^31^P environments. Data for (a) are taken from ref (29).

A deeper investigation into the structural differences was performed using ^6^Li and ^31^P NMR. The ^6^Li spectra given in Figure 5a are taken from previous work by Gautam et al. and show a shift with increasing disorder, which can be attributed to the expansion of the Li cage and occupancy of the non-T5 sites.^29^ The ^31^P NMR spectra, given in Figure 5b, show a distinct splitting, which is more pronounced for slow-cooled samples as well as prominent broadening of the peaks for the quench-cooled samples.

The splitting of the peaks is a result of the occupancy of the second coordination sphere. The phosphorus nuclei sit at the center of the PS_4_-tetrahedra and are sensitive to the chemical identity of the atoms they are bonded to as well as those in the second coordination sphere, which consists of the four 4*d* sites surrounding the tetrahedra (see Figure 5f). Although substitution of the sulfur in the PS_4_-tetrahedra result in shift differences of over 20 ppm, changes to the second coordination sphere result in differences of 2–8 ppm, giving rise to peak splitting.^9,27,65^ The five distinct peaks that emerge have been attributed to the five possible ways in which the 4*d* sites in the second sphere can be occupied, with four S atoms and no Br represented by 4S-0B, the most electron-poor environment and four Br atoms represented by 0S-4B. The other four possibilities (three S, one Br, etc.) sit in between (see Figure 5c,e).

The peak integral is a direct representation of the number of nuclei in the corresponding environment.^66^ Using the Br-occupancy at the 4*d* site obtained from XRD refinement, it is possible to calculate the theoretical random statistical probability distribution of the five environments using eq 5:^9,27^
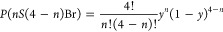
5

Here, P(*nS* (4 – *n*)Br) is the probability of obtaining the environment with *n* S atoms and (4-*n*) Br atoms, with *y* being the fraction of 4*d* sites occupied by *S*. The obtained integrals and theoretical distributions are given in Figure 5d,e. For both cooling methods, the distributions are close to random, showing no preference and a statistical distribution of S and Br over the 4*a* and 4*d* sites.

**Figure 6:**
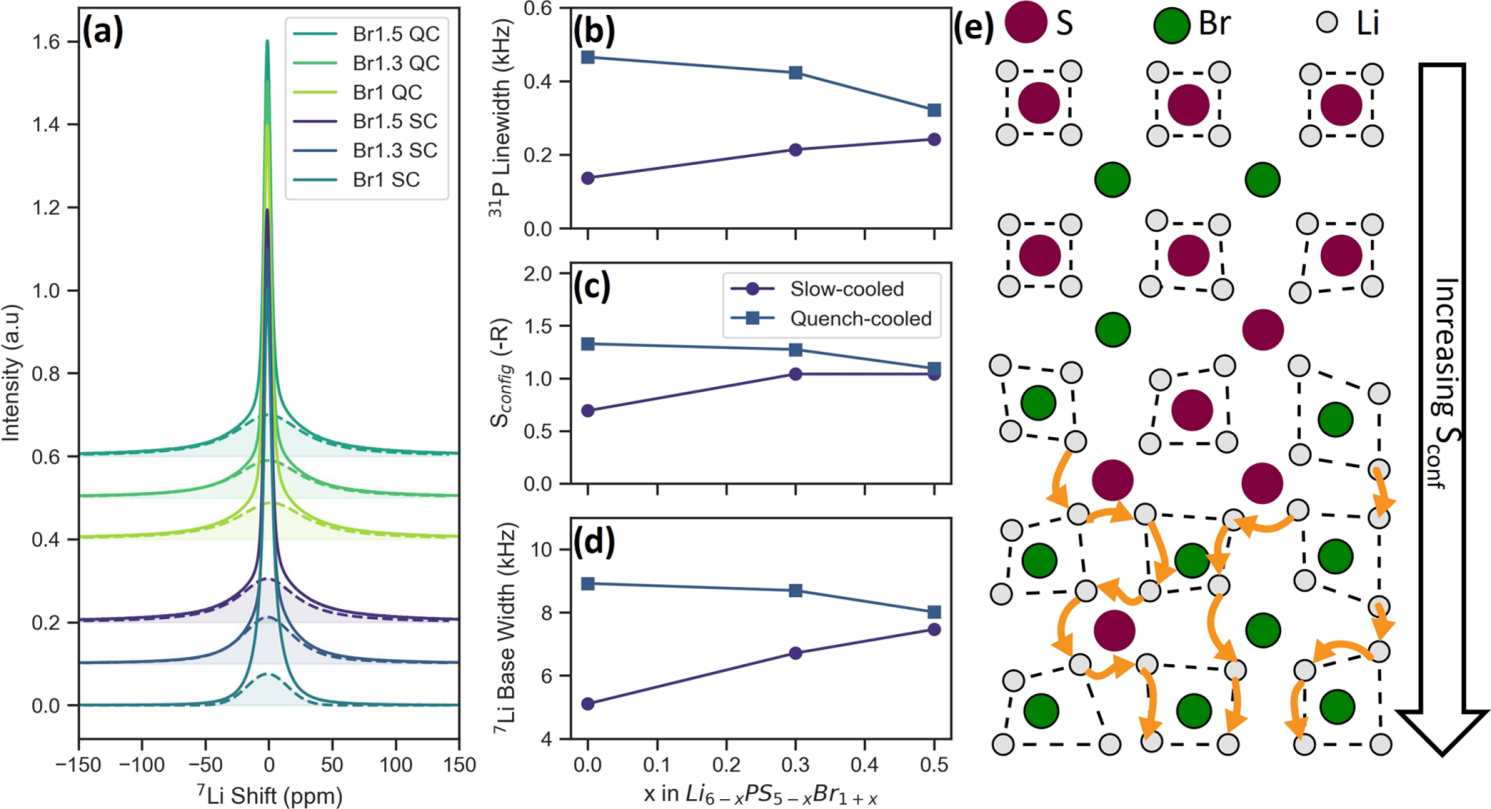
(a) Static ^7^Li spectra taken at 203 K, the broad base highlighted, (b) the ^31^P line width of the 2S-2B environment as a function of Br content at 293 K, (c) the calculated configurational entropy using eq 6, (d) the width of the base highlighted in (a), and (e) an illustration of the effect of increased configurational entropy, resulting in the distortion of lithium cages, the occupancy of non-T5 sites and increased percolation.

Although the 4*d* site disorder is able to explain the peak integrals, it cannot account for the differences in peak width. For the quench-cooled samples, the convoluted ^31^P peaks are significantly broader (see Figure 6b and SI Figure S12), indicating a higher degree of bond disorder, usually associated with Cl-based argyrodites.^6,21^ Besides the two coordination spheres mentioned, phosphorus is also sensitive to interactions with nuclei in the subsequent spheres, which include both the 4*d* and 4*a* sites. Although these interactions are not strong enough to induce peak splitting, a large degree of disorder within the entire anion sublattice can result in broadening of the peaks.

The configurational entropy can be used to quantify anion sublattice disorder through the ways in which Br and S can be placed on the 4*a* and 4*d* sites and is defined as^67^
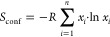
6

Here, *R* is the gas constant and *x_i_* represents the mole fraction of element *x* on sublattice *i* (see SI section 6). The result, shown in Figure 6c, mirrors the trend in peak line width observed.

The configurational entropy depends on the occupancy of the 4*d* and 4*a* site and is a measure of the disorder in the entire anion sublattice. An increase in anion sublattice disorder is predicted to benefit long-range Li diffusion in lithium argyrodite systems through distortion of the lithium environment.^12,30^ Lithium occupies cages with 4*d* sites at the center and 4*a* sites close to the corners. When all 4*d* sites are occupied by sulfur and all 4*a* sites by bromine, these cages are symmetric and close to the 4*d* site. This is the most energetically favorable position, minimizing interaction with neighboring lithium cations and making intercage movement unfavorable. Anion disorder, where both 4*a* and 4*d* sites are occupied by either element, results in distortion of these cages from this energetically favorable position as bromine is less electronegative than sulfur. Occupancy of non-T5 sites and intercage movement become more favorable, allowing for the formation of percolation pathways between cages (see Figure 6e).^12^ More anion sublattice disorder would result in more percolation pathways and hence increased Li diffusion.

Experimentally, the distortion in the Li sublattice would result in the observed shift in the ^6^Li spectra to lower ppm as the lithium cage expands and non-T5 sites are occupied (see Figure 5a) as well as a change in the quadrupolar coupling constant. Both isotopes of lithium (^6^Li and ^7^Li) are quadrupolar nuclei and hence sensitive to their environment’s symmetry. In an ideal argyrodite structure, the Li-occupied cages are symmetric. Distortion of these cages due to disorder breaks this symmetry.^68^ The sensitivity of a nucleus to this asymmetry is quantified by its quadrupolar moment. Although ^6^Li possess a rather small moment of −0.0808 fm^2^, ^7^Li has a larger moment of −4.01 fm^2^. In a ^7^Li static spectrum, the asymmetric environment would manifest as a broad base in the line shape at lower temperatures.^62^ The width of this base corresponds to the degree of asymmetry experienced by the lithium. Figure 6a shows the static solid-echo ^7^Li spectra at 203 K of the investigated samples. A broad bump at the base can be seen, indicative of a quadrupolar interaction. The peaks were deconvoluted to obtain the base widths, shown in Figure 6d, which follow the same trend as the configurational entropy.

From this, we see that the quench-cooling method increases 4*d* site disorder and disorder of the entire anion sublattice, quantified by eq 6. This results in a distortion of the lithium cages, which has been linked with an increase in intercage movement. Comparison between *S*_conf_ and the β value in Figure 3c shows a correlation between the increased anion sublattice disorder and a larger degree of intercage movement for the slow-cooled samples. For the quench-cooled samples, intercage movement occurs readily. However, for Br1 QC, both SLR and MD simulations show a larger degree of intercage movement compared with Br1.3 SC, which has a similar degree of site disorder. This could be attributed to it possessing the largest degree of anion sublattice disorder/highest *S*_conf_ which facilitates intercage movement despite a lower degree of site disorder. Furthermore, Br1 QC and Br1.3 QC possess similar values for *S*_conf_ and appear to display similar intercage jump rates, implying that except for Br1.5 QC, the *S*_conf_ correlates well with the degree of intercage movement. Regarding the long-range movement measured with EIS, we observe an increase with increasing *S*_conf_ for the slow-cooled samples but not for the quench-cooled samples.

From the structural information obtained via MAS NMR, we see that the site disorder on the 4*d* site alone cannot fully describe the structural changes observed. The configurational entropy takes the disorder of the entire sublattice into account and is able to describe the system more fully. Previous studies on Li argyrodites have noted a correlation between ^31^P peak width and increased *S*_conf_.^69^ Using the ^7^Li quadrupolar interaction, we are able to further link this to Li cage distortion as well. In previous studies on Li argyrodites, *S*_conf_ was varied through cationic and anionic doping, combined with slow cooling of the samples, and long-range techniques such as EIS and PFG NMR were used to probe lithium movement.^42,69,70^ By using two cooling methods, we are able to uncouple chemical and structural effects on *S*_conf_ and investigate both long-range and short-range movement by using EIS and SLR NMR, respectively. For the slow-cooled samples, we see an increase in long-range lithium movement, congruent with previous studies^42,69,70^ correlating with an increase in short-range movement. For the quench-cooled samples, there is a correlation between increasing short-range movement and increasing *S*_conf_. However, this correlation is lost for long-range movement. The difference in behavior between EIS and SLR warrants a comparison of these techniques.

### The Prefactor from EIS and NMR

The conductivity obtained from EIS follows eq 1, with *E*_a_ being the measurable activation energy barrier and σ_0_ a prefactor.^16^ Studies focusing on decreasing *E*_a_ often encounter the “prefactor dilemma” where a lower *E*_a_ also results in a lower prefactor and hence a lower conductivity.^71^ It can be argued that in the present case an increase in the prefactor with increasing site disorder results in a higher conductivity, even though *E*_a_ is increasing as well. The correlation between the activation barrier and the prefactor is termed the Meyer–Neldel rule and has been observed in conductivity studies for a variety of materials including semiconductors^72^ and ionic conductors.^48,73^ Various interpretations of the physical meaning of the Meyer–Neldel rule exist^74^ and it appears to be applicable to many processes that display Arrhenius-type behavior, including lithium-ion movement within solids.^75^ As both EIS and SLR NMR probe this movement, we will investigate and compare the results of both methods through the lens of Meyer–Neldel theory and attempt to correlate our observations with structural parameters.

The thermally activated jump rate (ν) of ions in a material can be described using an Arrhenius equation (eq 7) with Δ*G*_M_ being the migrational energy barrier, defined as the difference in free energy between the initial and saddle point.^76,77^
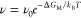
7

Δ*G*_M_ is defined as eq 8, with Δ*H*_M_ and Δ*S*_M_ being the enthalpy and entropy of migration, respectively,^78^ giving eq 9 for the jump rate.^79^
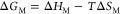
8
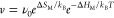
9

*E*_a_ from eq 1 can be further defined as consisting of the enthalpy of migration and the enthalpy of defect formation (Δ*H*_F_). The enthalpy of defect formation is close to negligible for most ionic conductors.^71,80^ The ionic conductivity was used to obtain the hopping frequency (ω) and Δ*H*_M_ (see SI section 7 for more details). Almond et al. argue that when Δ*H*_F_ is negligible, ω is the same as the hopping frequency in eq 7 and can be defined as eq 10.^81^
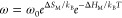
10

**Figure 7:**
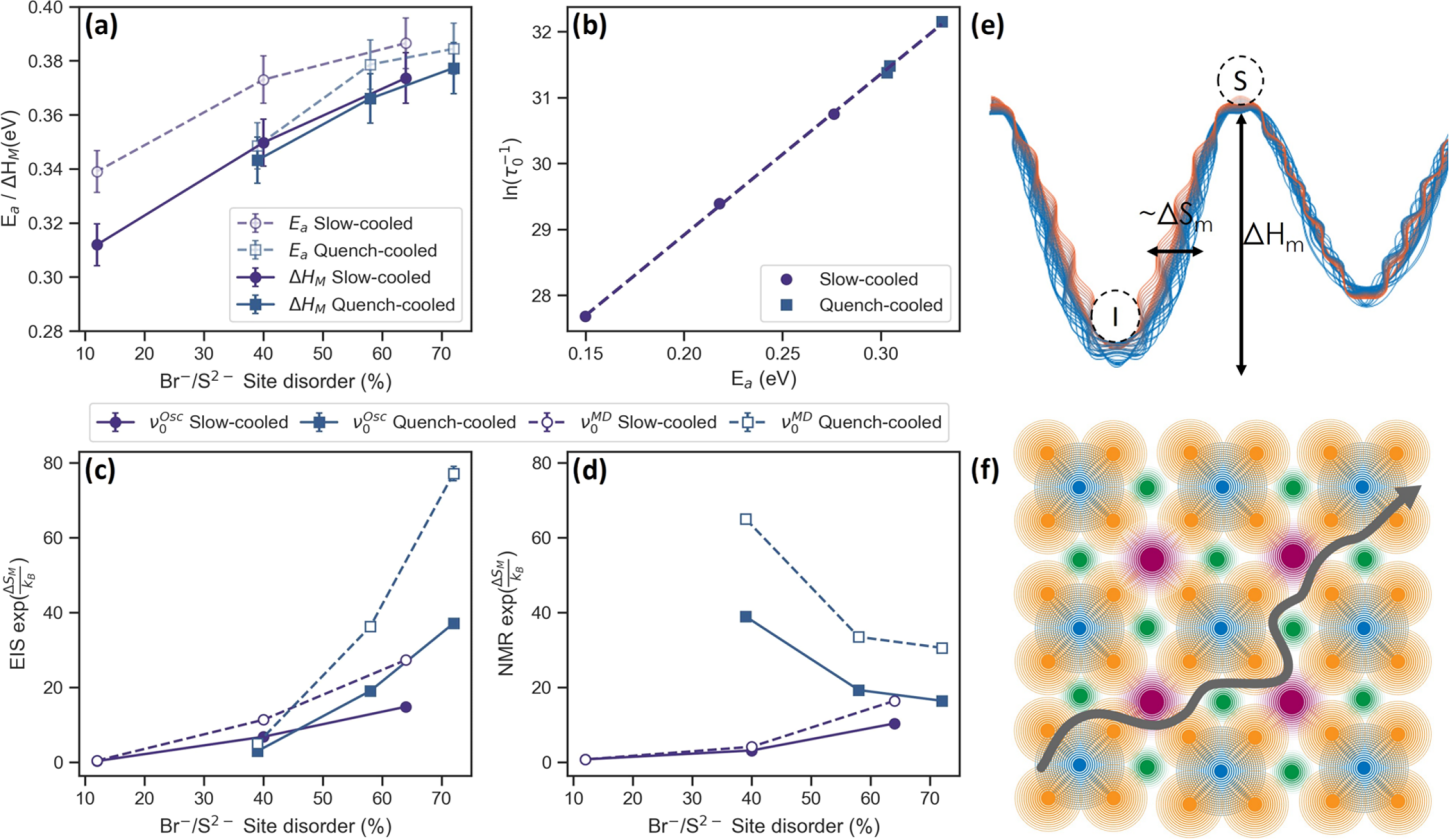
(a) Activation energy (*E*_a_) and enthalpy of migration (Δ*H*_M_) obtained from the EIS data; (b) the natural logarithm of the prefactor vs the activation energy obtained from the modified BPP fit, showing a linear correlation in line with the Meyer–Neldel rule; (c) entropy of migration (Δ*S*_M_) from the obtained the EIS data; (d) entropy of migration (Δ*S*_M_) from the obtained the BPP fit; (e) illustration showing the enthalpy of migration as the difference in energy between the initial (I) and saddle-points (S) and the entropy of migration as being proportional to the number of ways it can be surmounted; and (f) an illustration showing the crystal lattice as a collection of vibrating atoms allowing lithium movement through coupling to the phonon modes.

The difference between *E*_a_ and Δ*H*_M_, given in Figure 7a, is small, showing that Δ*H*_F_ is negligible (see Figure S8). By using the hopping frequency, we do not need to estimate the effects of the other terms in the equation describing conductivity (SI section 7). The obtained prefactor contains only an attempt frequency (ω_0_) and an entropic term .

A similar analysis can be performed with the data obtained from SLR. The prefactor from *T*_1_ SLR (τ_0_^–1^) is given in Figure 3d and is thought to be in the order of phonon frequencies of lithium ions in the material.^21,82^ It is expected to be between 10^12^ and 10^14^ Hz and has been shown to increase with decreasing lattice softness.^28,83^ Lattice softness refers to the polarizability of the lattice and hence the atoms within it. Larger, more polarizable atoms such as Br will form a softer lattice compared to smaller, harder atoms such as Cl.^28,83^ As more sulfur is substituted by bromine, lattice softness is expected to decrease, explaining the increase in τ_0_^–1^ for the slow-cooled samples in Figure 3d. Unexpectedly, for the quench-cooled samples, a slight decrease in the τ_0_^–1^ is observed. The difference in trend between the cooling methods hints at a stronger interplay with other structural parameters besides chemical composition. Plotting the natural logarithm of the NMR prefactors versus the activation energy reveals that they are linearly correlated (Figure 7b). This implies that the NMR jump process given by eq 2 follows the Meyer–Neldel rule and can be described by using eq 9 as well. The inverse of the correlation time (τ_c_) from eq 2 can be substituted for ν, giving:
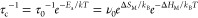



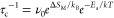
11

The experimentally obtained NMR prefactor can then be expressed as , consisting of an approximate attempt frequency term (ν_0_) and a term for the entropy of migration.^21,84,85^ To obtain the entropic term for EIS and SLR NMR, the experimentally obtained prefactors should be divided by an attempt frequency (ν_0_). Two methods for obtaining ν_0_ values were used and compared, namely, from MD simulations (ν_0_^MD^) or calculated by assuming a classical hopping mechanism based on eq 12 (ν_0_^Osc^)^86^ (see SI section 8 for more details). The results for EIS and NMR are given in Figure 7c,d, respectively, and show a clear difference in trends for the two techniques. It must be noted that for NMR, the prefactor describes both intercage and intracage jumps, and hence, the value for *a*_0_ used in eq 12 could be overestimated, resulting in a smaller value for ν_0_ and hence an underestimation of Δ*S*_M_. However, both ν_0_^MD^ and ν_0_^Osc^ give the same trends.
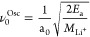
12

The entropy of migration has been interpreted in various ways, with the two most prominent interpretations being the multiexcitation entropy model (MEE) and the phonon vibrational model.^87−89^

Yelon *et al.* argue that when the energy barrier for a jump (Δ*H*_M_) is larger than ℏ*ω* (the energy of available excitations) and *k*_B_*T*, multiple excitations must work together to surmount it.^49,79^ The higher this barrier, the more excitations are needed and hence the more ways there are of combining them. The entropic term (Δ*S*_M_) can therefore be defined as the number of ways to assemble the necessary amount of excitations needed (*n* = Δ*H*_M_/ℏω) to pass the barrier from the total number possible (*N*) in the interaction volume, resulting in eq 13, which can be simplified when assuming that *n* ≪ *N* (see Figure 7e).
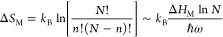
13

The Δ*S*_M_ values obtained from NMR and EIS are shown in Figure 7c,d, respectively, exhibiting a clear difference in trends yet somewhat similar values. Both follow the trends observed for the Δ*H*_M_ values obtained from the respective techniques (for NMR, these are the *E*_a_ values given in Figure 3b), which are congruent with eq 13.

For NMR, there is a difference in Δ*S*_M_ values between the cooling methods used, mirroring the trend seen for *S*_conf_ (Figure 6c). *S*_conf_ is a measure of the distortion of the lithium cage. A more distorted cage results in more lithium ions occupying less energetically favorable positions, destabilizing the initial state. This could result in more states being available to assemble the necessary excitations and, hence, a larger *N* value in eq 13.

For EIS, the Δ*S*_M_ values do not correlate with *S*_conf_, correlating with the 4*d* site disorder/Br content instead. Discrepancies between NMR and EIS have been observed before for activation energies and are ascribed to correlation effects and the difference in the length scale probed (see SI Section 11 for a rough calculation).^90−92^ NMR is sensitive to movement occurring within the time frame of the Larmor frequency (116 MHz) and is hence more local, whereas EIS is sensitive to long-range movement.^93^ This difference in length scale also affects the prefactors obtained and hence the entropy of migration.^94^

The second interpretation, from which eq 14 is derived, is related to the phonon vibrations of the materials and is often used to relate the Δ*S*_M_ values obtained from EIS to material structure and chemical composition.^28,95−97^ Here, Δ*S*_M_ is the ratio of the product of the normal frequencies of the lattice where lithium is at the initial site (ν_*i*_^*I*^) and the lattice where lithium is at the saddle point (ν_*i*_^*S*^). The attempt frequency ν_0_ is seen as the frequency of the vibrational mode that carries lithium across the saddle point. This equation relates the entropy of migration to the lattice vibrations experienced by the lithium ion during diffusion.^98^
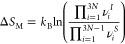
14Although determination of the components of eq 14 is challenging, various relationships have been found between the vibrational characteristics of the lattice and the prefactor obtained from EIS and AIMD.^98^ Elemental substitution is the most common method to alter lattice softness, as quantified by the Debye frequency. Various studies have found that the addition of “softer”, larger, more polarizable elements such as I in Li_6_PS_5_X (X = Cl, Br, I) results in a decrease in the Debye frequency along with a decrease in the EIS prefactor and activation energy.^28,95−97^ Although this explains the general trend in Δ*S*_M_ observed in Figure 7d, the difference between the two cooling methods implies that chemical composition alone does not describe the lattice vibrations experienced by lithium fully. Gelin et al. performed a computational study on the diffusion of ions in crystalline silicon and germanium, showing that movement of many atoms along the diffusion path results in the alteration of the phonon mode spectrum of the material, with lower vibrational modes becoming more enhanced. Enhancement of these soft modes results in a higher Δ*S*_M_.^89^ Xu et al. have shown that for sulfide-based lithium-ion conductors, coupling between lithium ions and low-medium frequency modes, specifically those associated with the sulfur and bromine surrounding the intercage bottleneck is seen and results in an enhancement of the soft phonon modes as well.^34^ They argue that coupling of lithium ions with these modes benefits long-range movement, as it allows lithium to oscillate with larger amplitude in the direction of bottlenecks. This was later also observed computationally for Li_6_PS_5_Cl.^99^ Furthermore, previous NMR studies on argyrodite systems have found a correlation between the coupling of lithium- and PS_4_-tetrahedral motion around the bottleneck between lithium cages and increased ion conductivity obtained from EIS for Li_6_PS_5_X (X = Cl, Br, I) with the degree of coupling depending on the lattice parameter and the degree of site disorder.^56,61,100^ Hence it is possible that the difference in Δ*S*_M_ values obtained from EIS stems from the degree of coupling between lithium and the PS_4_ tetrahedra. Spin relaxation *T*_1_ NMR is sensitive to local movement and hence the ratio of intracage and intercage jumps. As EIS is sensitive to only long-range movement, the coupling between ions and the lattice vibrations associated with low-frequency modes becomes more pronounced.

## Conclusions

In this study, the effects of increasing Br content (*x* = 0, 0.3, 0.5) and using two synthesis cooling methods (slow-cooling and quench-cooling) were explored using EIS, ^7^Li *T*_1_ SLR NMR, and ^6^Li, ^7^Li, and ^31^P NMR, allowing us to disentangle chemical composition and site disorder.

- Using ^7^Li *T*_1_ SLR NMR, we are able to probe the short-range lithium dynamics of the Li_6–*x*_PS_5–*x*_Br_1+*x*_-series. For slow-cooled samples, an increase in Br content/site disorder results in an increase in intercage movement. For the quench-cooled samples, intercage movement occurs readily for all samples and an increase in Br content/4*d* site disorder lowers the energy barrier for intercage movement. The same is observed in MD simulations of the systems.
- The chemical environment was probed with ^6^Li, ^7^Li, and ^31^P NMR. The ^31^P MAS NMR line width reflects the degree of site disorder across the entire anion sublattice and was found to be described well by the configurational entropy (*S*_conf_) of this sublattice. The distortion of the Li cage can be quantified with the ^7^Li static NMR base width, being a measure of the quadrupolar interaction. This distortion correlates with the ^31^P line width and *S*_conf_, showing that increase in anion sublattice disorder/*S*_conf_ results in increased Li cage distortion. In addition, increase in *S*_conf_ correlates with increase in intercage movement seen via ^7^Li *T*_1_, congruent with theoretical predictions.^12^ Additionally for the slow-cooled samples, an increase in *S*_conf_ results in increase EIS conductivity in agreement with previous studies.^42,69,70^ For quench-cooled samples, this is not the case. Hence, we conclude that an increase in *S*_conf_ through chemical doping results in increased EIS conductivity, whereas an increase in *S*_conf_ through quenching does not, indicating that the correlation between configurational entropy and conductivity is intricate.
- The prefactor obtained from ^7^Li *T*_1_ NMR obeys the Meyer–Neldel law, allowing extraction of the migration entropy Δ*S*_M_ and comparison to values obtained from EIS. We find that the Δ*S*_M_ obtained from NMR correlates well with *S*_conf_ and hence the degree of cage distortion for both cooling methods. On the other hand, the Δ*S*_M_ obtained from EIS shows a correlation with *S*_conf_ for the slow-cooled samples only. There is a correlation between Δ*S*_M_ and the 4*d* site disorder and chemical composition. This implies that the chemical composition and soft phonon modes resulting from the interaction between lithium and the 4*d* site are more important for long-range diffusion.

Our results highlight the importance of the synthesis method on the chemical structure and analysis of the materials. By changing the cooling method from slow cooling to quenching, the relationship between structural descriptors (site disorder or *S*_conf_) and short-range and long-range diffusion is changed completely. The implicit assumption that improved short-range diffusion automatically results in improved long-range conductivity is challenged, and the importance of chemical composition and ion-phonon interactions are highlighted. We observe that an increase in site disorder results in an increase in EIS conductivity and corresponding activation energy, regardless of the cooling method used. However, we observe that the cooling method has a profound effect on short-range lithium movement probed by *T*_1_ SLR NMR, that goes beyond 4*d* occupancy.

## Supporting Information Available

EIS data processing, calculation of the 4d and diffusion distances, calculation of configurational entropy, as well as a brief description of migrational entropy (Figures S1–S13) (PDF)
