# Supplementary material for: Decoding Structural Disorder, Synthesis Methods, and Short- and Long-Range Lithium-Ion Transport in Lithium Argyrodites (Li6–xPS5–xBr1+x)
Source: Chem Mater. 2025 Jan 29;37(3):869–83. doi: 10.1021/acs.chemmater.4c02010 (PMC11823417; doi:10.1021/acs.chemmater.4c02010)
Supplement: Supplementary file 1 — cm4c02010_si_001.pdf [file cm4c02010_si_001.pdf]

Supporting Information

Decoding Structural Disorder, Synthesis Methods, and Short- and Long-Range Lithium-Ion Transport in Lithium Argyrodites ($Li_{6-x}PS_{5-x}Br_{1+x}$)

Hanan Al-Kutubi, Ajay Gautam, Anastasia K. Lavrinenko, Alexandros Vasileiadis, Jouke R. Heringa, Swapna Ganapathy* and Marnix Wagemaker*

Section Storage of Electrochemical Energy, Department of Radiation Science and Technology, Faculty of Applied Sciences, Delft University of Technology, Mekelweg 15, 2629 JB, Delft, The Netherlands

Table of Contents

1 Results obtained by Gautam et al. 3
2 The Neldel-Meyer Energy 5
3 Distances calculated from dipolar coupling 5
4 The Br-occupancies on 4d- and 4a-site 6
5 The probability density of Li^+ obtained from MD-simulations 7
6 Configurational entropy 8
7 EIS Data processing 8
8 Calculation distances 4d-sites 10
9 The ^{31}P MAS NMR Linewidths 11
10 A Brief explanantion of migrational entropy 12
11 Calculating the diffusion distances 13
12 References 15

1 Results obtained by Gautam et al.

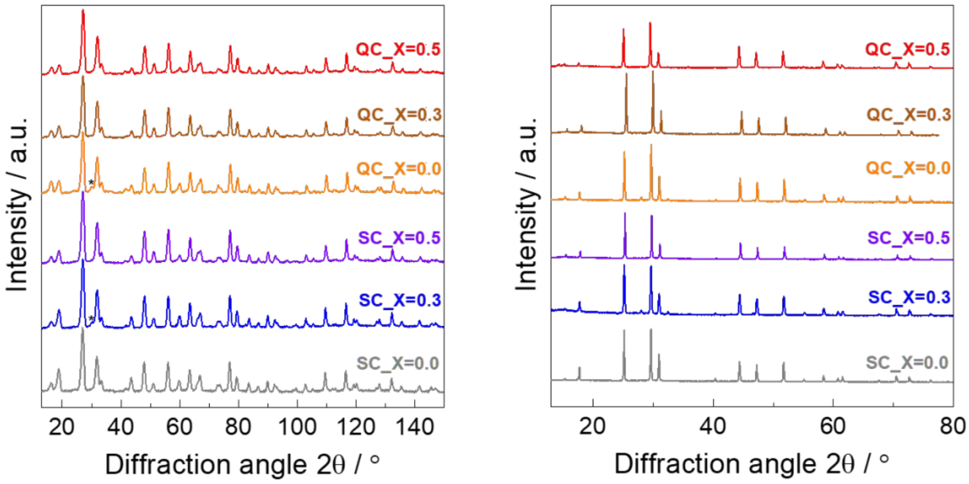

Figure S1. The Neutron (left) and X-ray (right) diffraction patterns obtained for slow-cooled (SC) and quench-cooled (QC) samples. Reused with permission from A. Gautam et al. Chem. Mater. *2023,* 35*, 8081 (ref 29). Copyright 2023 American Chemical Society.*

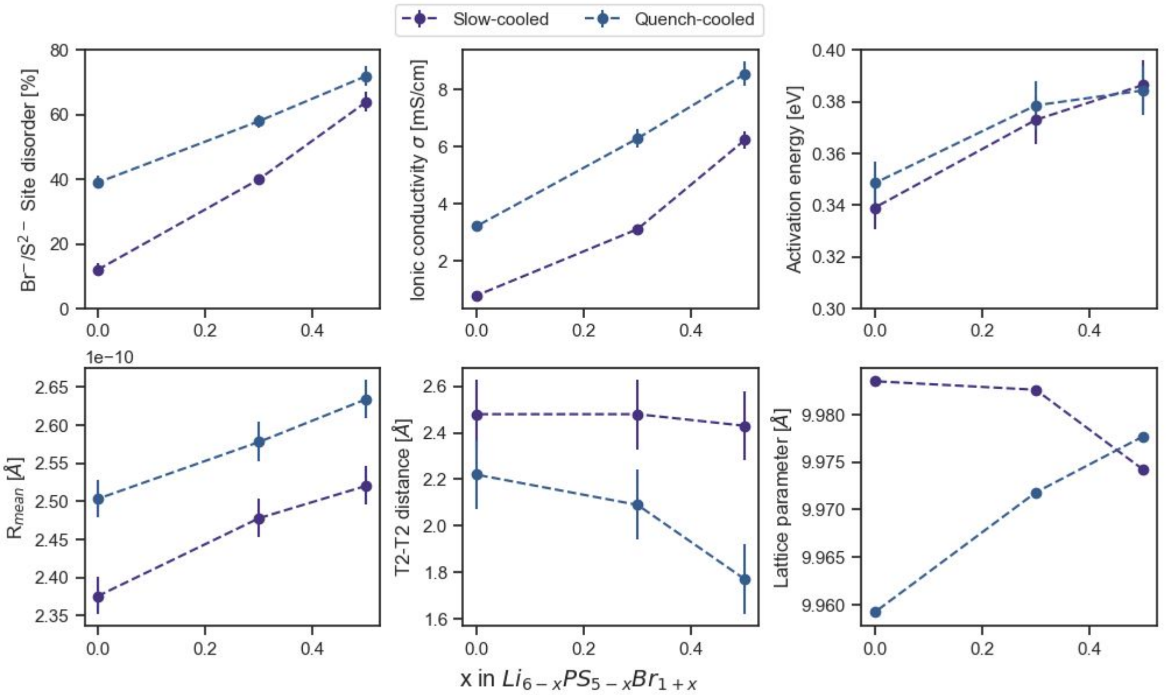

Figure S2. Results obtained by Gautam et al[1] showing as a function of Br-content (a) the 4d site disorder, (b) the ionic conductivity from EIS, (c) the activation energy obtained from EIS, and the structural parameters obtained from XRD and neutron diffraction including (d) the radius of the Li-cage, (e) the T2-T2-distance and the lattice parameter (f) . The data is taken from ref 1.

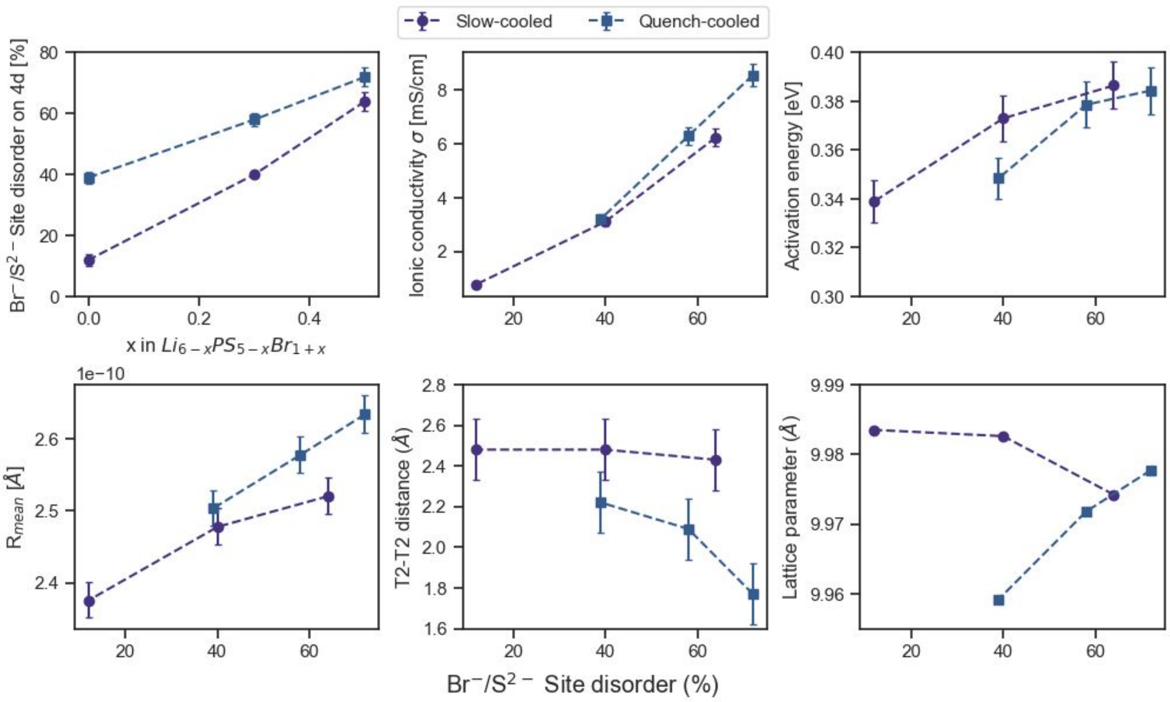

Figure S3. Results obtained by Gautam et al[1] showing as a function of site-disorder (a) the 4d site disorder, (b) the ionic conductivity from EIS, (c) the activation energy obtained from EIS, and the structural parameters obtained from XRD and neutron diffraction including (d) the radius of the Li-cage, (e) the T2-T2-distance and the lattice parameter (f) . The data is taken from ref 1.

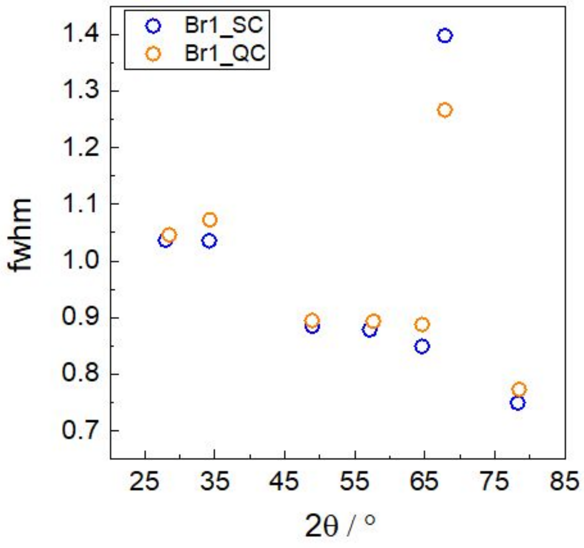

Figure S4. The full-width half maximum obtained from X-ray diffraction for Br1 SC and Br1 QC as a function of diffraction angle. Reused with permission from A. Gautam et al. Chem. Mater. *2023,* 35, *8081 (ref 1). Copyright 2023 American Chemical Society.*

2 The Neldel-Meyer Energy

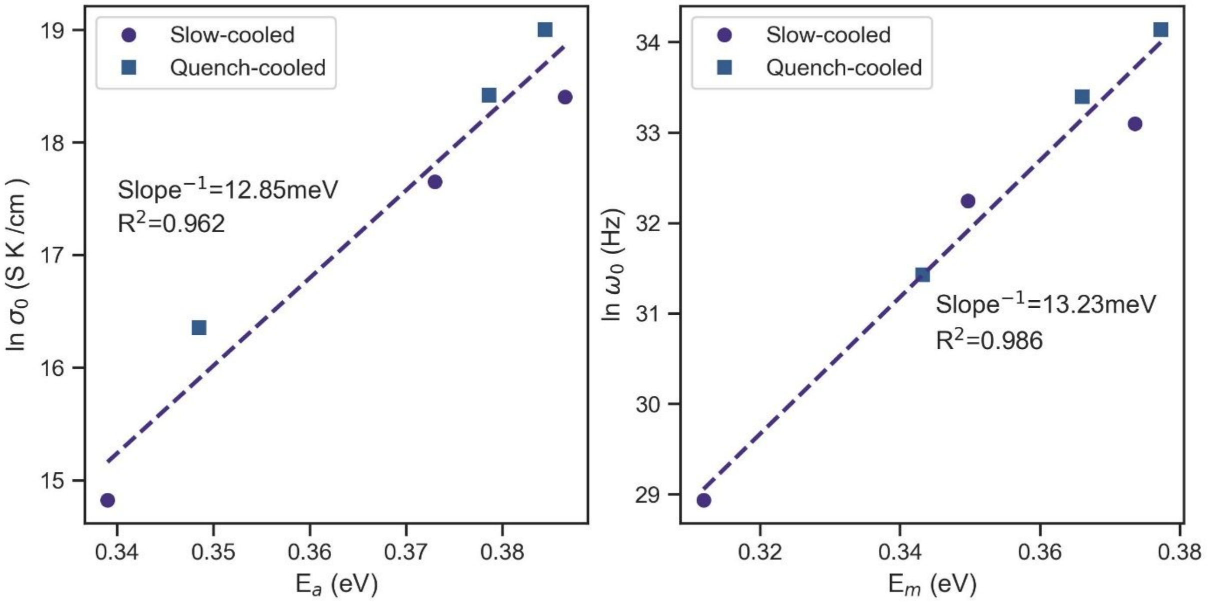

Figure S5. The prefactor versus the activation energy from EIS (left)[1] and hopping (right) and the inverse of the slope in meV corresponding to the Meyer-Neldel energy. Data for the right panel is taken from ref 1.

3 Distances calculated from dipolar coupling

The distances were calculated using the formula,[2] where γ_{Li} is the ^{7}Li gyromagnetic ratio, $\hbar$ the reduced Planck constant, μ_0 the vacuum permeability and C the constant obtained from the modified BPP-model fit.

$$r = \sqrt[6]{\frac{3\gamma_{Li}^4 \hbar^2 \mu_0^2}{32\pi^2\, C}}$$

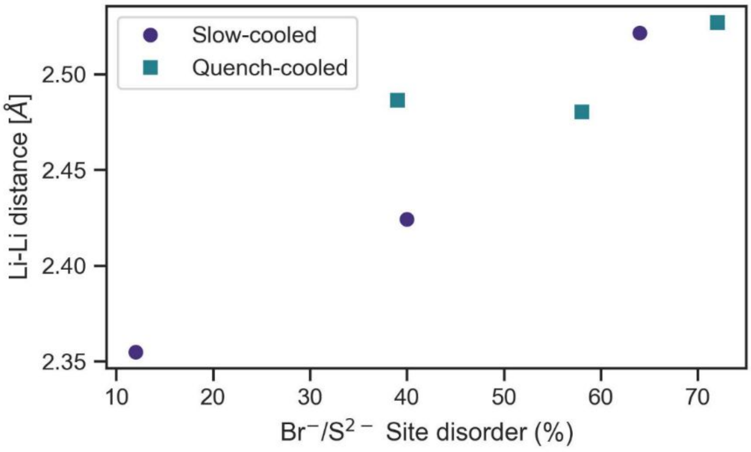

Figure S6. The Li-Li distance calculated assuming exclusively homonuclear dipolar coupling as a function of site-disorder

4 THE BR-OCCUPANCIES ON 4D- AND 4A-SITE

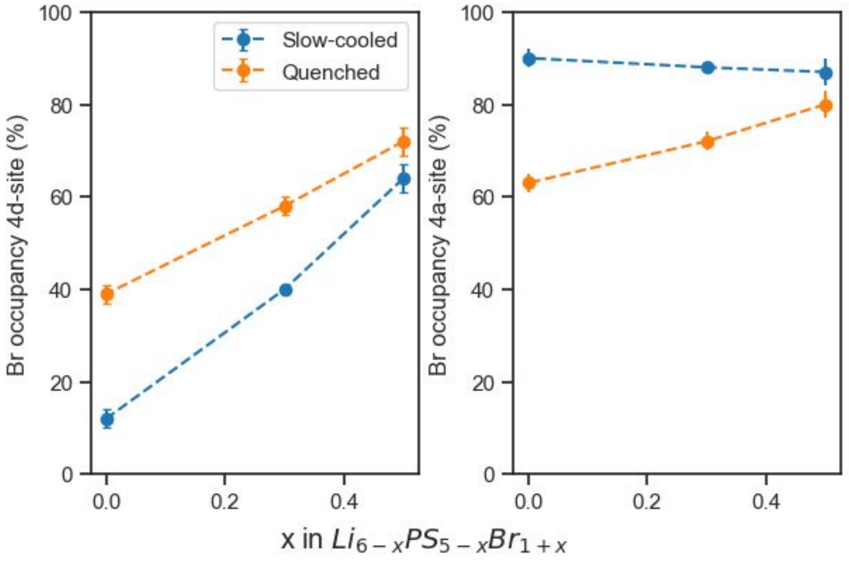

Figure S7. Plots of the percentage of 4d-sites (left) and 4a-sites (right) occupied by bromine (Br) as a function of Br-content for the slow-cooled and quenched samples. The sulphur occupancy is defined as 100%-Br occupancy. Data is taken from ref 1.

5 THE PROBABILITY DENSITY OF Li^+ OBTAINED FROM MD-SIMULATIONS

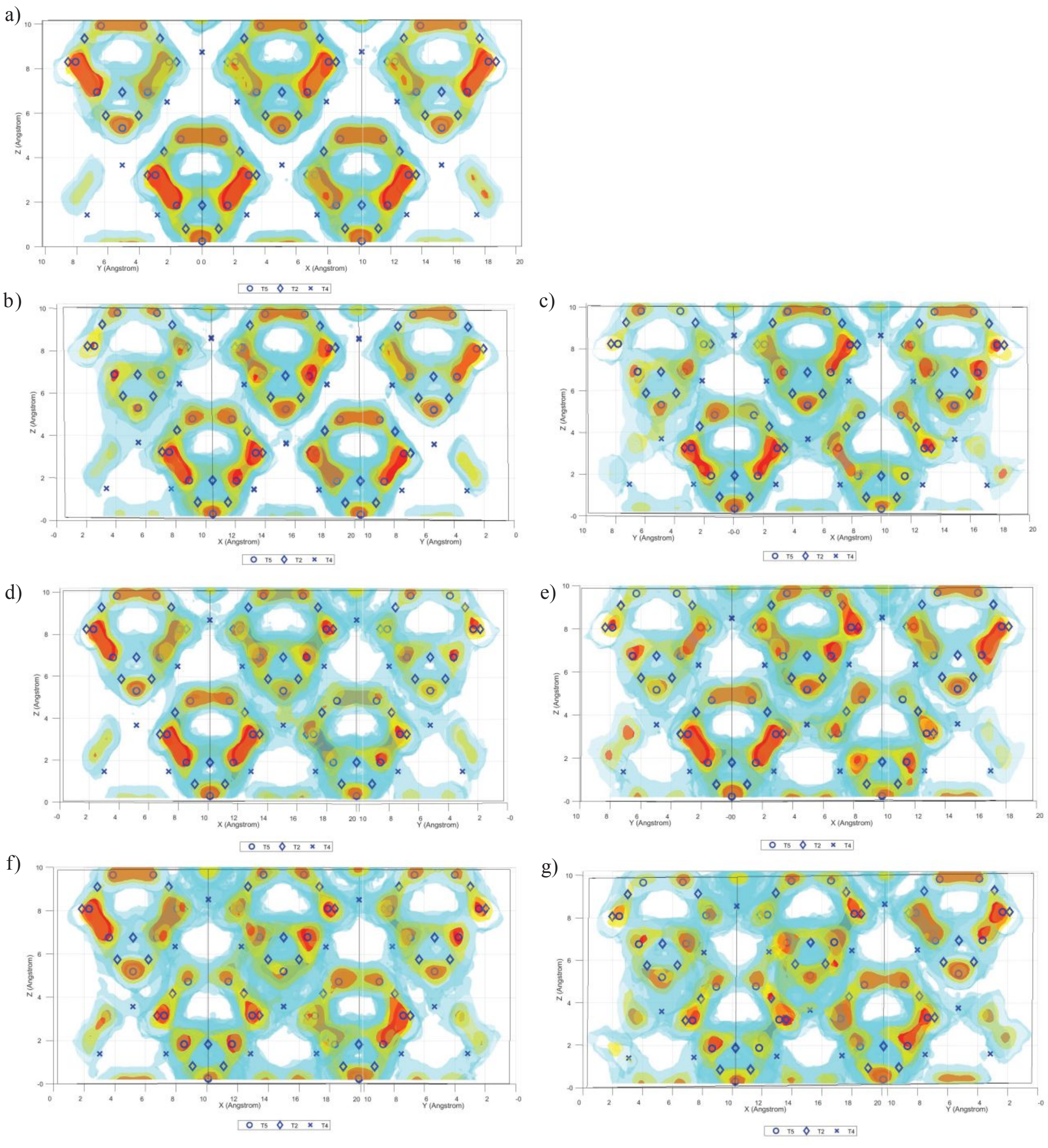

Figure S8. The probability density of Li^+: a) Ordered structure; b) Li_6PS_5Br slow cooled; c) Li_6PS_5Br quench cooled; d) $Li_{5.7}PS_{4.7}Br_{1.3}$ slow cooled; e) $Li_{5.7}PS_{4.7}Br_{1.3}$ quench cooled; f) $Li_{5.5}PS_{4.5}Br_{1.5}$ slow cooled; g) $Li_{5.5}PS_{4.5}Br_{1.5}$ quench cooled.

6 CONFIGURATIONAL ENTROPY

The configurational entropy was calculated using[3]:

$$S_{conf} = -R \sum_{i=1}^{n} x_i \cdot \ln x_i$$

Two sublattices were considered in this system, namely the 4a- and 4d- sublattices. For each sublattice, two elements were considered, namely sulphur(S) and bromine (Br). Using the occupancy of bromine and sulphur at the 4d- and 4a-sites as the x_i-values, the configurational entropy can be defined as:

$$S_{conf} = -R[Br_{4d}\ln(Br_{4d}) + S_{4d}\ln(S_{4d}) + Br_{4a}\ln(Br_{4a}) + S_{4a}\ln(S_{4a}) +]$$

7 EIS DATA PROCESSING

The obtained EIS data was processed to obtain the hopping frequency (ω) using the method of Almond et.al.[4] and further developed by Li et al.[5]

The ionic conductivity can be defined as:

$$\sigma T = Ne^2a^2c(1-c)\gamma k_B^{-1}\,\omega_p$$

with N being the number of lattice sites available for the mobile ion, e the electronic charge, a the jump distance, c the fractional occupancy of mobile sites, γ a correlation factor and a hopping frequency (ω_p here or ω in the main text).[6]

The angular frequency dependant conductivity (σ_ω) can be defined as:

$$\sigma_\omega = \sigma_0 + A\omega^n$$

with σ_0 being the conductivity in the d.c. limit and ω the angular frequency. Almond et. al. also derive that

$$A = \frac{\sigma_0}{\omega_p^n}$$

Combining these equations gives us

$$\sigma_\omega = \sigma_0\left[1 + \left(\frac{\omega}{\omega_p}\right)^n\right]$$

Li et al show that ω_p can be obtained from the frequency-dependent conductivity spectra when

$$\sigma_\omega = 2\sigma_0$$

In the Bode spectra obtained from EIS, this corresponds to the frequency at which the resistance is half of the d.c. limit resistance. By assuming that the system can be described as a resistance and a constant phase element in parallel, the obtained impedance can be expressed as[7]

$$Z(\omega) = \frac{R}{1 + (i\omega)^\alpha QR}$$

Where R is the resistance of the electrolyte, Q is the CPE-constant and α is the dimensionless constant between 0 and 1 that represents the deviation from ideal capacitor behaviour. By assuming that $Z(\omega_p) = 0.5\ R$

we get: $\omega_p = \left(\frac{1}{QR}\right)^{\alpha}$

Obtaining the Q-, R- and α-values from the impedance fit allows us to obtain ω_p in rad/s for all samples at the temperatures given. These values were checked manually at 223 °K using the Bode-plot obtained.

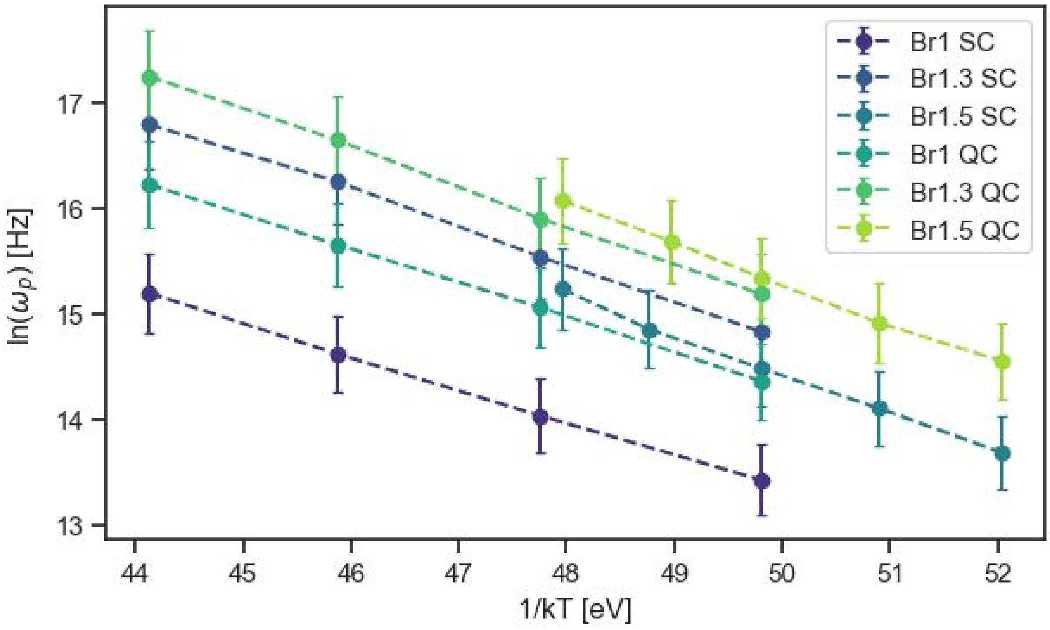

Figure S9. The natural logarithm of the jump frequencies obtained from EIS-analysis vs. 1/kT for all six samples. The dashed lines are a guide for the eye.

Finally, using: $\omega = \omega_0 e^{\frac{\Delta S_M}{k_B}} e^{\frac{-\Delta H_M}{k_B T}}$

we can obtain ΔH_M and the entropic pre-factor. Note the conversion from rad/s to Hz.

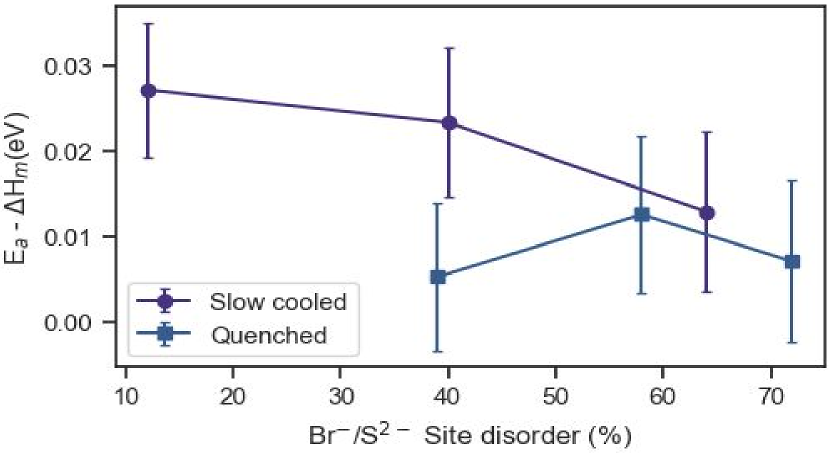

Figure S10. The difference between the activation energy (E_a) and the enthalpy of migration (ΔH_m) obtained from EIS.

8 CALCULATION DISTANCES 4D-SITES

The formula for calculating ν_0^{Osc} is:

$$\nu_0^{Osc} = \frac{1}{a_0}\sqrt{\frac{2E_a}{M_{Li^+}}}$$

Here, M_{Li^+} is the mass of the lithium ion and E_a the activation energy obtained from the BPP-fit from SLR NMR or the ΔH_M-value from EIS.[29]

The a_0 is defined as the distance between two 4d-sites within the unit cell (see Figure S8a) minus the R_{mean}.

We know from symmetry that $b_1 + b_2 = a_0$

The distance between two 4d-sites at the centre of the Lithium cages (a) was calculated based on the cell constant (c) obtained using the formula :$a_0 = \frac{1}{2}\sqrt{c^2 + c^2} - 2R_{mean} = \frac{\sqrt{2}}{2}c - 2R_{mean}$

The values for c and R_{mean} were obtained from Gautam et. al.[1]

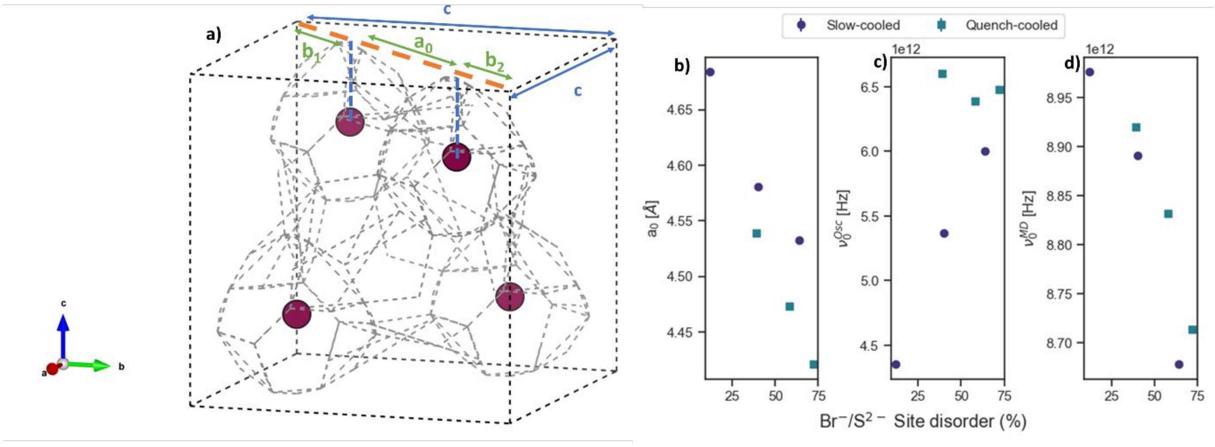

Figure S11. (a) Argyrodite lattice showing only the 4d-site positions and Li-cage outlines and the definitions for c, a_0, b_1 and b_2, (b) the a_0 values obtained, (c) the ν_0^{Osc}-values used in the main text and (d) the ν_0^{MD}-values obtained from MD-simulations and used in the main text.

9 THE ^{31}P MAS NMR LINEWIDTHS

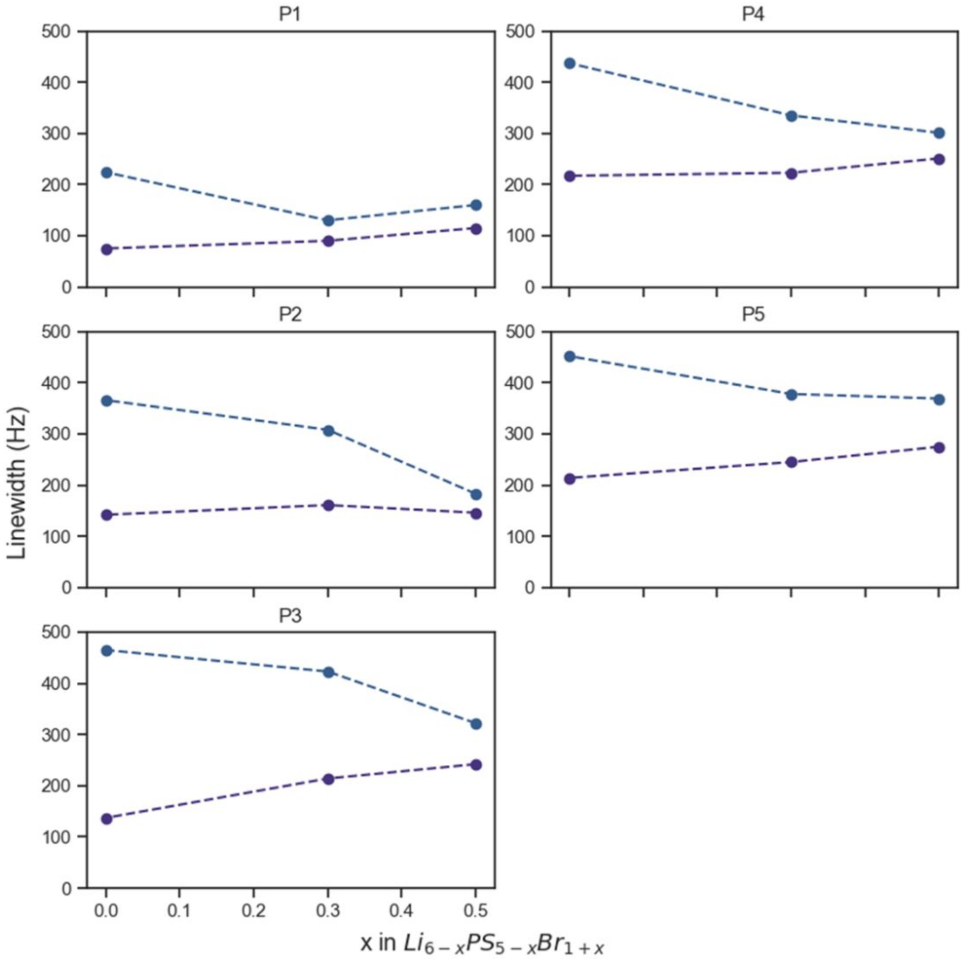

Figure S12. The MAS linewidths of the 31P environments mentioned in the main text at 293 K.

10 A BRIEF EXPLANANTION OF MIGRATIONAL ENTROPY

Classically, the entropy of a system can be described using the Boltzmann entropy equation (Equation 14). This equation gives the relationship between the entropy of a system and W, the number of ways in which microscopically distinct states can give rise to the same macroscopic thermodynamic state.[8]

$$S = k_B \ln(W) \; (Eq. 14)$$

The entropy of migration has been interpreted in various ways, with the two most prominent interpretations being the multi-excitation entropy model (MEE) and the phonon vibrational model. [9–11] To understand the relationship between this term and material structure, both interpretations will be discussed here.

The multi-excitation entropy model (MEE) provides a similar interpretation of ΔS_M to the classical one and assumes the existence of multiple excitations. Yelon et al. argue that when the energy barrier for a jump (ΔH_M) is larger than $\hbar\omega$ (the energy of available excitations) and $k_B T$, multiple excitations must work together to surmount it.[8,12] The higher this barrier is, the more excitations are needed and hence the more ways there are of combining them. The entropic term (ΔS_M) can therefore be defined as the number of ways to assemble the necessary amount of excitations needed (n) to pass the barrier from the total number possible (N) in the interaction volume, resulting in Equation 15, which can be simplified when assuming that n<<N (see **Error! Reference source not found.**e). Assuming that for similar compounds the energy per excitation is similar, a higher ΔS_M-value can have two origins according to Equation 15. Either the energy barrier is larger (larger ΔH_M) or a larger number of excitations is available (larger N).

$$\Delta S_M = k_B \ln\left[\frac{N!}{n!(N-n)!}\right] \sim k_B \frac{\Delta H_M \ln N}{\hbar\omega} \; (Eq. 15)$$

The second interpretation is related to the phonon vibrations of the materials and is often used to relate the ΔS_M-values obtained from EIS to material structure and chemical composition. [13–16] Phonons describe the collective vibrations of atoms in a lattice and depend on the lattice parameters as well as the chemical composition. Phonon frequencies increase for harder, smaller, less polarizable atoms and more spatial constriction. The relationship between the entropy of migration and the phonon vibrations of the lattice is given by Equation 16. [17,18] Here, ΔS_M is the ratio of the product of the normal frequencies of the lattice where lithium is at the initial site (ν_i^I) and the lattice where lithium is at the saddle point (ν_i^S). The attempt frequency ν_0 is seen as the frequency of the vibrational mode that carries lithium across the saddle point. This equation relates the entropy of migration to the lattice vibrations experienced by the lithium ion during diffusion.[19]

$$\Delta S_M = k_B \, ln\left(\frac{\prod_{i=1}^{3N} \nu_i^I}{\prod_{i=1}^{3N-1} \nu_i^S}\right) \; (Eq. 16)$$

11 CALCULATING THE DIFFUSION DISTANCES

The root mean square displacement of a particle, assuming three dimensional Brownian motion can be expressed as[20] :

$$x = \sqrt{6Dt}$$

With D being the diffusion constant in [cm^2/s] and t being the time over which diffusion takes place in seconds.

For impedance spectroscopy, the relationship between conductivity and the is:

$$\sigma = \frac{D_{EIS} n e^2 z^2}{kT}$$

giving:

$$D_{EIS} = \frac{\sigma kT}{n e^2 z^2}$$

Here, σ is the conductivity in [S/cm], k is the Boltzmann constant, T is the temperature in [K], e is the elementary charge (1.602x10^{-19} C), z is the ionic charge (equal to 1 for Li^+) and n is the charge carrier density of lithium ions, which is defined as:

$$n = \frac{4(6 - x)}{c^3}$$

Where the nominator is the amount of Li-ions per unit cell and c is the lattice constant in [cm].

For the time t, we take the inverse of maximum frequency of the EIS experiment (1/10^6 Hz).

For NMR, the diffusion constant is defined as:

$$D_{NMR} = \frac{a^2}{K\tau_c}$$

With a being the jump distance in cm taken as the distance between two 4d-sites defined in section 8, K being a dimensionality constant equalling 6 for 3D diffusion and τ_c being the NMR correlation time in seconds defined as Equation 1 in the main text.

Using the above equations and the values derived from EIS and NMR, we obtain the following values for the D and MSD. Note that for the diffusion constant from EIS, k is in [J/K], whereas for NMR it is in [eV/K].

The equation for D_{NMR} is the same as that for x, with t being τ_c and x the intercage distance. Hence we also calculate the distance x by taking using D_{EIS}. The distances are approximations but show that the diffusion distance for EIS is about 10x as large as that of NMR.

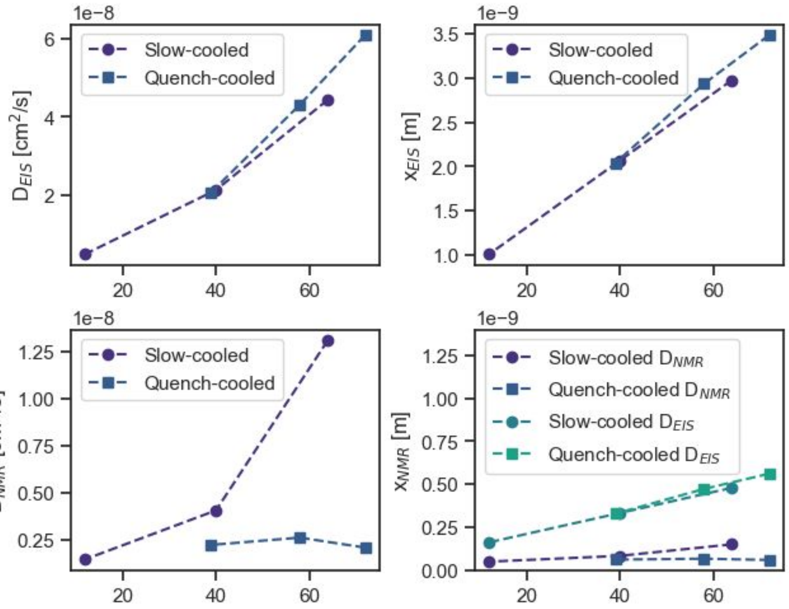

Figure S13. The diffusivity (a) and diffusion distance (b) for EIS and the diffusivity calculated using T_1 (c) and diffusion distances from D and D_{EIS} (d). The time t is $1/10^6$ for EIS and $1/1.6*10^8$ for NMR

12 REFERENCES

1. Gautam, A., Al-Kutubi, H., Famprikis, T., Ganapathy, S. & Wagemaker, M. Exploring the Relationship Between Halide Substitution, Structural Disorder, and Lithium Distribution in Lithium Argyrodites (Li6–xPS5–xBr1+x). *Chem. Mater.* **35**, 8081–8091 (2023).
2. Duff, B. B. *et al.* Toward Understanding of the Li-Ion Migration Pathways in the Lithium Aluminum Sulfides Li3AlS3 and Li4.3AlS3.3Cl0.7 via 6,7Li Solid-State Nuclear Magnetic Resonance Spectroscopy. *Chem. Mater.* **35**, 27–40 (2023).
3. Mollaei, Z. *et al.* Configurational entropy as a simple input data for glass science and engineering. *Materials Today Communications* **32**, 104153 (2022).
4. Almond, D. P., Hunter, C. C. & West, A. R. The extraction of ionic conductivities and hopping rates from a.c. conductivity data. *J Mater Sci* **19**, 3236–3248 (1984).
5. Li, X. *et al.* Hopping Rate and Migration Entropy as the Origin of Superionic Conduction within Solid-State Electrolytes. *J. Am. Chem. Soc.* **145**, 11701–11709 (2023).
6. Goodenough, J. B. Review Lecture - Fast ionic conduction in solid. *Proceedings of the Royal Society of London. A. Mathematical and Physical Sciences* **393**, 215–234 (1997).
7. Chang, B.-Y. Conversion of a Constant Phase Element to an Equivalent Capacitor. *J. Electrochem. Sci. Technol* **11**, 318–321 (2020).
8. Yelon, A., Movaghar, B. & Crandall, R. S. Multi-excitation entropy: its role in thermodynamics and kinetics. *Rep. Prog. Phys.* **69**, 1145 (2006).
9. Almond, D. P. & West, A. R. The activation entropy for transport in ionic conductors. *Solid State Ionics* **23**, 27–35 (1987).
10. Nowick, A. S., Lee, W.-K. & Jain, H. Survey and interpretation of pre-exponentials of conductivity. *Solid State Ionics* **28–30**, 89–94 (1988).
11. Gelin, S., Champagne-Ruel, A. & Mousseau, N. Enthalpy-entropy compensation of atomic diffusion originates from softening of low frequency phonons. *Nat Commun* **11**, 3977 (2020).

12. Yelon, A., Movaghar, B. & Branz, H. M. Origin and consequences of the compensation (Meyer-Neldel) law. *Phys. Rev. B* **46**, 12244–12250 (1992).

13. Kraft, M. A. *et al.* Influence of Lattice Polarizability on the Ionic Conductivity in the Lithium Superionic Argyrodites Li6PS5X (X = Cl, Br, I). *J. Am. Chem. Soc.* **139**, 10909–10918 (2017).

14. Chen, R. *et al.* Influence of Structural Distortion and Lattice Dynamics on Li-Ion Diffusion in Li3OCl1–xBrx Superionic Conductors. *ACS Appl. Energy Mater.* **4**, 2107–2114 (2021).

15. Krauskopf, T. *et al.* Comparing the Descriptors for Investigating the Influence of Lattice Dynamics on Ionic Transport Using the Superionic Conductor Na3PS4–xSex. *J. Am. Chem. Soc.* **140**, 14464–14473 (2018).

16. Bernges, T., Culver, S. P., Minafra, N., Koerver, R. & Zeier, W. G. Competing Structural Influences in the Li Superionic Conducting Argyrodites Li6PS5–xSexBr (0 ≤ x ≤ 1) upon Se Substitution. *Inorg. Chem.* **57**, 13920–13928 (2018).

17. Vineyard, G. H. Frequency factors and isotope effects in solid state rate processes. *Journal of Physics and Chemistry of Solids* **3**, 121–127 (1957).

18. Huntington, H. B., Shirn, G. A. & Wajda, E. S. Calculation of the Entropies of Lattice Defects. *Phys. Rev.* **99**, 1085–1091 (1955).

19. Muy, S., Schlem, R., Shao-Horn, Y. & Zeier, W. G. Phonon–Ion Interactions: Designing Ion Mobility Based on Lattice Dynamics. *Advanced Energy Materials* **11**, 2002787 (2021).

20. Yu, C. *et al.* Facile Synthesis toward the Optimal Structure-Conductivity Characteristics of the Argyrodite Li6PS5Cl Solid-State Electrolyte. *ACS Appl. Mater. Interfaces* **10**, 33296–33306 (2018).
